# Influenza A/Hong Kong/156/1997(H5N1) virus NS1 gene mutations F103L and M106I both increase IFN antagonism, virulence and cytoplasmic localization but differ in binding to RIG-I and CPSF30

**DOI:** 10.1186/1743-422X-10-243

**Published:** 2013-07-25

**Authors:** Samar K Dankar, Elena Miranda, Nicole E Forbes, Martin Pelchat, Ali Tavassoli, Mohammed Selman, Jihui Ping, Jianjun Jia, Earl G Brown

**Affiliations:** 1 Department of Biochemistry, Microbiology and Immunology, Faculty of Medicine, University of Ottawa, 451 Smyth Rd, Ottawa, Ontario K1H 8M5, Canada; 2 Emerging Pathogens Research Centre, Faculty of Medicine, University of Ottawa, 451 Smyth Rd, Ottawa, Ontario K1H 8M5, Canada; 3 Chemistry, University of Southampton, Southampton SO17 1BJ, UK; 4 Cancer Sciences, Faculty of Medicine, University of Southampton, Southampton SO16 6YD, UK

**Keywords:** NS1, Nonstructural protein 1, Influenza A virus, Host switch, Nuclear, Cytoplasmic localization, Nuclear export signal, Host gene expression, Virulence, Interferon, H7N9, H5N1, H6N1, Alveoli, tropism

## Abstract

**Background:**

The genetic basis for avian to mammalian host switching in influenza A virus is largely unknown. The human A/HK/156/1997 (H5N1) virus that transmitted from poultry possesses NS1 gene mutations F103L + M106I that are virulence determinants in the mouse model of pneumonia; however their individual roles have not been determined. The emergent A/Shanghai/patient1/2013(H7N9)-like viruses also possess these mutations which may contribute to their virulence and ability to switch species.

**Methods:**

NS1 mutant viruses were constructed by reverse genetics and site directed mutagenesis on human and mouse-adapted backbones. Mouse infections assessed virulence, virus yield, tissue infection, and IFN induction. NS1 protein properties were assessed for subcellular distribution, IFN antagonism (mouse and human), CPSF30 and RIG-I domain binding, host transcription (microarray); and the natural prevalence of 103L and 106I mutants was assessed.

**Results:**

Each of the F103L and M106I mutations contributes additively to virulence to reduce the lethal dose by >800 and >3,200 fold respectively by mediating alveolar tissue infection with >100 fold increased infectious yields. The 106I NS1 mutant lost CPSF binding but the 103L mutant maintained binding that correlated with an increased general decrease in host gene expression in human but not mouse cells. Each mutation positively modulated the inhibition of IFN induction in mouse cells and activation of the IFN-β promoter in human cells but not in combination in human cells indicating negative epistasis. Each of the F103L and M106I mutations restored a defect in cytoplasmic localization of H5N1 NS1 in mouse cells. Human H1N1 and H3N2 NS1 proteins bound to the CARD, helicase and RD RIG-I domains, whereas the H5N1 NS1 with the same consensus 103F and 106M mutations did not bind these domains, which was totally or partially restored by the M106I or F103L mutations respectively.

**Conclusions:**

The F103L and M106I mutations in the H5N1 NS1 protein each increased IFN antagonism and mediated interstitial pneumonia in mice that was associated with increased cytoplasmic localization and altered host factor binding. These mutations may contribute to the ability of previous HPAI H5N1 and recent LPAI H7N9 and H6N1 (NS1-103L+106M) viruses to switch hosts and cause disease in humans.

## Introduction

The 1997 highly pathogenic avian influenza (HPAI) H5N1 (prototype A/HK/156/1997 (H5N1)) and 2013 low pathogenic avian influenza (LPAI) H7N9 (prototype A/Shanghai/patient 1/2013 (H7N9)) lineages of influenza A virus (IAV) have both acquired all 6 of their internal protein encoding genome segments including the NS1 gene through reassortment with the A/Beijing/1/1994(H9N2) (BJ1994) lineage of viruses in China [1-3]; and both cause fatal interstitial pneumonia in humans [4-6]. Early and recent isolates of the LPAI H9N2 lineage viruses are pathogenic for mice without adaptation [7-9] and have directly infected humans in 1999 in Hong Kong (see [10]). Although the majority of human cases of H5N1 and H7N9 avian IAV are associated with direct contact with infected poultry, without sustained human-to-human transmission, they continue to pose a pandemic threat [11]. The scope and nature of this threat is undefined because the genetic basis for host switching and virulence is largely unknown. The evolution of an engineered H5N1 IAV laboratory strain on serial nasal passage in ferrets that is capable of airborne-transmission after acquiring 5 mutations [12] suggests that further adaptation in humans may generate a transmissible form of human H5N1 or possibly H7N9. It is therefore necessary to determine the genetic determinants of H9N2 IAV origin genes in virulence that are relevant to interspecies transmission.

In this paper we address the characterization of functional determinants of virulence in the 1997 H5N1 NS1 gene that contribute to host switching and virulence. We have previously shown that the pair of mutations, F103L + M106I, in the A/Hong Kong/156/1997 (H5N1) NS1 gene (H5N1-NS1) controlled virulence on the A/PR/8/1934 (H1N1) (PR8) virus backbone [13]; (where PR8 has the structural protein functions required to infect alveoli and cause interstitial pneumonia [14]). F103L and M106I mutations have been independently selected in mammals, on mouse adaptation of human A/HK/1/68 (H3N2) (HK) virus, and shown to possess adaptive gain-of-function properties *in vitro* (transcription and replication) and *in vivo* (increased virulence and IFN antagonism in the mouse lung) but with reduced ability to bind the cleavage and polyadenylation specificity factor 30Kd subunit (CPSF30) [15]. The role of the NS1 103L and 106I mutations relative to the consensus residues 103F and 106M is controversial because other studies have shown reduced virulence associated with a loss of ability to bind CPSF and antagonize IFN induction in A/HK/483/1997 (H5N1) [16], in contrast to the same mutations in the A/HK/156/1997 (H5N1) NS1 gene on the PR8 backbone that increases IFN antagonism and replication associated with increased virulence [13]. Thus these mutations appear to be subject to epistasis where their phenotypes are dependent on the presence of other mutations, as has been shown to be prevalent in IAV surface proteins [17,18] and reported in the NP gene [19]. This is consistent with evolutionary studies of RNA viruses which show that their genetically simple and compact genomes demonstrate extensive interactions, or epistasis among and within multifunctional proteins, where one mutation may enhance one function to the detriment of another function but which is dependent on other mutations (reviewed [20]). For example HIV protease gene inhibitor resistant mutants acquire mutations sequentially and in defined orders, where the first confers drug resistance and subsequent mutations compensate for side-effects of the first mutations but which are deleterious when analyzed in isolation [21]. Consequently, it is necessary to consider the effects of genomic context when studying viral evolution.

A major function of NS1 is its involvement in modulating multiple host responses including antagonism of the type I IFN response (reviewed [22]). This is achieved through interfering with pathogen associated molecular pattern (PAMP) receptor interactions. NS1 binds the viral PAMPS, dsRNA and ssRNA, [23,24] to block recognition and signaling by cytoplasmic retinoic inducible gene (RIG-I) and surface expressed toll-like receptors (TLR-3 and TLR-7) [25-27]. NS1 therefore blocks IFN induction at the pre-transcriptional level to prevent activation of the transcription factors IRF-3, IRF-7, ATF-2/c-Jun and NF-κB that signal IFN induction [28-30]. Another strategy employed by NS1 is the direct binding to RIG-I [31] as well as TRIM25 [32] and RIPLET (human and mouse RIG-I E3 ubiquitin ligases respectively) resulting in inhibition of the RIG-I/IPS-1 mediated activation of IRF-3 and NF-κB [33]. Mouse cells lack TRIM25 interaction but possess RIPLET binding by NS1 protein to inhibit ubiquitination and signaling by RIG-I [34]. The A/WSN/1933 NS1 protein has been shown to bind the CARD and regulatory domain (RD) of RIG-I but not the helicase domain in a bacterial reverse two hybrid assay (RTHS) [35].

NS1 inhibits the 3′-end processing of host mRNAs, including IFN-β and IFN responsive genes, by binding to the CPSF30-F2F3 (F2F3 zinc finger domain of CPSF-30) [36,37], PABPNI [38] and U6 snRNA [39], therefore down regulating host mRNA splicing and maturation. In addition to preventing the maturation of the poly-adenylated cellular mRNAs, NS1 also sequesters them in the nucleus by directly binding of mRNA [40,41] or indirectly via mRNA protein factor binding [38,42].

NS1 blocks IFN induced signalling functions in the cytoplasm [43] and cytoplasmic IFN effectors, 2′-5′ OAS and PKR to support viral gene expression [44-46]. Mutations at positions 103 and 106 in PR8 NS1, (F103S and M106I), have been shown to function to increase cytoplasmic localization in MDCK cells, which may contribute to increased cytoplasmic functioning [47].

NS1 uses the host translation machinery to selectively enhance viral protein translation and therefore replication [48,49]. NS1 F103L and M106I mutations also increase viral RNA polymerase activity in the luciferase mini-genome assay and increase transcription in infected mouse cells [15]. NS1 has also been shown to interact with all IAV proteins including all 3 viral polymerase subunits in 2-hybrid assays [50]; and host RNA helicase A (RHA) to enhance viral polymerase activity [51].

IAV virulence is multigenically controlled [52,53]; however genetic studies have identified specific NS1 residues with virulence. Previous studies of pathogenic avian A/HK/156/97 H5N1 showed that the NS1 gene mediated increased virulence in the mouse [54] and swine models [55] when analyzed on the PR8 backbone. Furthermore other studies of virulence identified single mutations in the NS1 gene (P42S, D92E and V149A) as well as multiple mutations in the PDZ ligand domain that increased virulence, replication and reduced IFN protein levels [55-58]. A naturally occurring 5 aa deletion of NS1 residues 191–195 of H5N1 swine viruses contributed to reduced pathogenicity in mice and chicken that was associated with reduced NS1-CPSF30 binding and IFN antagonism [59].

In this paper we demonstrate the ability of both the F103L and M106I mutations to increase replication, alveolar tropism and virulence in the poultry adapted human isolate A/HK/156/97 (H5N1) NS1 gene on the PR8 backbone. We show that each of these mutations increases cytoplasmic localization in mouse cells but that they differ in their effects on binding to the CPSF30 as well as human RIG-I domains.

## Materials and methods

### Cells

Mardin-Darby canine kidney (MDCK) cells, human embryonic kidney cells (293T), monkey kidney cells (Vero) and mouse kidney epithelial cells (M1) were maintained as described previously [53].

### Viruses

Recombinant influenza viruses on the A/PR/8/1934(H1N1) or A/HK/1/68 (H3N2) backbone were synthesized by reverse genetics as previously described [60,61]. The NS genes of A/Hong Kong/156/97 (H5N1) (A/HK/156/97) (Genbank AF036360) (synthesized by Biomatic Corporation, Cambridge, Ontario) and A/HK/1/68 (H3N2) were cloned into the pLLB(A) bidirectional vector by homologous recombination as described [60,61]. The A/Puerto Rico/8/34 (A/PR/8/34) NS gene and genome in the pHW2000 bidirectional plasmid was obtained from R. Webby, (St. Jude Children’s Research Hospital, Memphis). Site directed mutagenesis was achieved by polymerase chain reaction to introduce point mutations into the A/HK/1/68 (H3N2), A/PR/8/34 (H1N1) and H5N1 NS genes by using the primer pairs indicated in Additional file 1: Table S1 using 50 ng of the each NS gene of interest and the following conditions: 1 cycle (98°C for 30 sec), 30 cycles (98°C for 10 sec, 52°C for 30 sec, 72°C for 2 min 30 sec) and 1 cycle (72°C for 8 min). PCR products were treated with 1 unit of the Dpn1 (New England BioLabs, Pickering, ON) for 1 hour at 37°C to remove the input plasmid. Each of the NS plasmid constructs along with the 7 plasmids encoding the viral structural genes of A/PR/8/34 (H1N1) or A/HK/1/68 (H3N2) in pLLB were used to generate recombinant viruses as previously described [13,15]. The NS gene sequence of the rescued viruses was verified by sequence analysis. Viruses were grown in 10 day old specific pathogen free (SPF) embryonated chicken eggs (Canadian Food Inspection Agency, Ottawa, Ontario). Viruses were titrated by plaque assay in MDCK cells as described previously [13].

### CPSF-30 F2F3-FLAG expression plasmid

Synthesis of the CPSF-30 F2F3-FLAG tagged fragment clone in the pET17 vector under the control of the T7 promoter was previously described [15].

### Assessment of replication of viruses in mouse lungs

Groups of 19–21 gram, female, Swiss-Webster CD-1 mice (from Charles River Laboratories, Montreal, Quebec, Canada) were infected intranasally under halothane anesthesia with 5 × 10^3^ pfu of each of the viruses in a volume of 50 μl [62]. CD-1 mice were sacrificed at 1, 3, 5 and 7 days post infection (dpi) and lungs from three mice were collected for each day. Organs were weighed, diluted 1:4 in PBS and homogenized by bead mill (Mixer Mill MM 200, Retsch, Germany) for quantification by plaque assay.

### Assessment of replication of viruses in cells

M1 cells were infected in triplicate with the indicated viruses at moi = 2 with sampling of supernatant to measure IFN-β and infectious yield at 24 hpi by ELISA and plaque assay, respectively, in duplicate as described previously [13]. Vero cells were similarly infected in triplicate at moi = 0.01 with analysis of virus yield by plaque assay at 24 and 48 hpi, but with 0.5 μg TPCK trypsin/ml in the medium.

### Mouse survival assay

Groups of 5 female, 19-21 g, BALB/c or Swiss-Webster (CD-1) mice were infected intranasally with 50 μL of defined virus dosages under halothane anesthesia. Mortality was monitored for 10 dpi. Body weight was recorded daily and the LD_50_ was calculated by the Karber-Spearmen method [63].

### Animal ethics approval

This study was carried out in accordance and compliance with the guidelines of the Canadian Council on Animal Care (CCAC) as described earlier [15]. The protocol was approved by the University of Ottawa Animal Care Committee (Protocol Number: BMI-85).

### Immunofluorescence staining of CD-1 lungs

CD-1 mice were infected intranasally with 1 × 10^5^ pfu of each of the recombinant viruses and lungs were collected 3 dpi respectively. Lungs were inflated and fixed with 3.7% formaldehyde and frozen sections were stained as described previously [13]. Following washing, the slides were incubated with 100 μl of the primary rabbit anti-PR8 antibody (pre-adsorbed with mouse lung extract, used at 1:500) at 4°C overnight. Images were taken at 20× magnification using an Olympus BX50 microscope and processed in a parallel manner using Photoshop 7.0.

### Luciferase assay

The reporter plasmids expressing firefly luciferase under the control of IFN-β (p125) and IRF-3 human promoters (p55C1B) (Obtained from Yan Zhou, University of Saskatchewan) were used to measure the relative ability of NS1 proteins to suppress transfection-induced IFN-β and IRF-3 promoted luciferase induction. A second reporter plasmid expressing *Renilla* luciferase under the control of the constitutive simian virus 40 promoter (pRL-SV40) was included as an internal transcription control. Plasmid transfections (n=3) were performed in 293-T human embryonic kidney cells as previously described [53]. Briefly, 20 ng of firefly reporter plasmids, 20 ng of pLLB NS (or empty pLLB vector as a negative control) plasmids and 10 ng of the internal control pRL-SV40 were mixed with lipofectamine 2000 transfection reagent (Invitrogen, Burlington, Ontario) and incubated for 30 min at RT before application to wells in a 96 well poly-D-lysine-treated plates. Sixteen hours post transfection, the previous transfection mix on cells was removed and the cells were transfected with 1.0 ug of synthetic analog of dsRNA, polyinosine-polycytosine (Poly I:C) (Amersham Pharmacia Biotech) to induce the IFN-β promoter. At 40 h post-transfection, luminescence was measured according to the Promega Dual-Glo Luciferase Assay System protocol, using a Glomax Multi-Detection System Model 9301–010 (Fisher Scientific, Nepean, Ontario). Relative luciferase activities were calculated as the average +/− standard deviation of the ratios of firefly and renilla luciferase luminescence for three biological replicates. Uniform NS1 protein expression was demonstrated by western blot for NS1 and actin as described previously [10].

### NS1-CPSF30-F2F3 binding

The NS1 and CPSF-30-F2F3 expression and binding assays were performed as described previously [15]. The H5N1-NS-103L+106I (wt) plasmid as well as the mutants H5N1-NS-L103F+106I, H5N1-NS-103L+I106M and H5N1-NS-L103F+I106M gene segments were cloned in the pLLB vectors under the control of the T7 promoter. Briefly, 0.5 μg of the various NS plasmids as well as FLAG-tagged CPSF-F2F3 (FLAG-F2F3) fragment were expressed *in vitro* using TnT T7 Quick-Coupled Transcription/Translation System (Promega) in the presence of 10 μCi ^35^S-methionine and cysteine (Express ^35^S Protein Labeling Mix, Perkin Elmer). In an α-FLAG pulldown assay, equal volume of the *in vitro* translated and ^35^S-labeled H5N1-NS and the three mutant NS proteins were mixed with FLAG-F2F3 or H_2_O as a control (Additional file 1: Figure S2) and precipitated using Protein G Dynabeads (Invitrogen, Burlington, Ontario) pre-incubated with α-FLAG M2 antibody (Sigma, St Louis, Missouri). Following washing beads were analyzed by SDS-PAGE electrophoresis and autoradiography.

### NS1 protein localization

Monolayers of M1 cells were infected with the rPR8-H5N1-NS-103L+106I (n=3) as well as the three mutants (rPR8-H5N1-NS-L103F+106I, rPR8-H5N1-NS-103L+I106M and rPR8-H5N1-NS-L103F+I106M (n=2)) at MOI=2 and compared to mock infected cells. Sixteen hours post infection the cell supernatant was discarded and the whole cell lysate was collected in NP40 lysis buffer for the whole cell fraction, as well as fractionation into cytoplasmic and nuclear fractions by centrifugation [64] followed by SDS PAGE and western blotting probed for NS1 (polyclonal rabbit serum), NP (polyclonal rabbit serum), M1 (polyclonal rabbit serum), histone H3 (rabbit anti-histone H3, CT, pan clone A35; Millipore, Billerica, MA, USA) and β-tubulin (mouse anti-tubulin α; Sigma, Burlington, ON). Antibodies were detected by incubation with HRP conjugated goat anti-rabbit or goat anti-mouse antibody (Sigma, Burlington, ON) followed by SuperSignal West Pico chemiluminescent substrate (Pierce, Thermo Fisher Scientific, Nepean, ON) and exposure to X-ray film.

### Human RIG-I domain binding using bacterial reverse two-hybrid system (RTHS)

Each RTHS bacterial strain was constructed to contain the chromosomally incorporated NS1 proteins and each RIG-I domain (CARD, helicase and RNA binding regulatory domain (RD)) as previously described [35]. Using the RTHS, two hybrid-binding results in inhibition of selectable gene expression that inhibits replication (depicted in Additional file 1: Figure S1). For drop spotting each RTHS strain were grown overnight with shaking, at 37°C in LB medium with Ampicillin (50 μg/mL) and Spectomycin (25 μg/mL). The following day 20 μl of culture was diluted in 10% glycerol solution and ten-fold serial dilutions prepared in a sterile 96-well plate for each strain. 2.5 μl of each dilution was drop-spotted onto selective plates (200 ml Agar, 25 ml minimal media, 10 ml of 50% glycerol, 250 μl 1 M MgSO_4_, 50 μg/mL Ampicillin and 25 μg/mL Spectomycin), containing 25 μg/mL Kanamycin, 2.5 mM 3-amino-1,2,4-triazole and increasing levels of IPTG (10 μM, 25 μM, 50 μM and 100 μM), which induces the expression of the target proteins. Replication was observed after 2 days and colonies counted and plotted relative to IPTG concentration.

### Microarray analysis

We used primary microarray data from infected M1 cells compared to mock-infection with PBS for cells infected with HK-wt as well as NS1 mutants F103L, M106I and F103L + L106I (and M106V as another reference adaptive mutation at position 106 [15]) in infected M1 cells (MOI = 2 at 8 hpi), n = 3 biological replicates and analyzed them independently here using the methods described previously (Forbes et al. submitted). We also analyzed human gene transcription relative to mock PBS infected controls for the HK-wt (103F + 106M) and NS1 mutants F103L, M106I, M106V and the double F103L + L106I mutant using A549 cells infected at MOI = 2 with collection of total RNA at 8 hpi. At 8 hpi, the cellular RNA was collected using manufacturer’s protocol (QIAGEN RNeasy mini-kit) quantified using a PowerWave XS2, Microplate Spectrophotometer (BioTek Instruments, Inc. Winooski, VTUnited States ), and ribosomal RNA quality and integrity was confirmed using an Agilent 2100 Bioanalyzer (Agilent Technologies Canada Inc. Mississauga, Canada). The microarray analysis was performed by StemCore Laboratories (Ottawa, Ontario, Canada) using the Genechip Mouse Exon 1.0 ST Array and the Genechip Human Exon 1.0 ST Array for the respective mouse and human samples (Affymetrix, Santa Clara, CA, USA). Genes were considered as up or down regulated relative to mock infected cells if they were ≤1 or ≥1 log2 fold different (≤ or ≥ 2 fold differences) expression level and were statistically significant at the p ≤ 0.05 level using ANOVA, and further analyzed using Bioconductor software packages in R for clustered heat maps of gene expression and mutants, Pheatmaps, (pheatmap_0.7.4.tar.gz) analyzed using the complete linkage and Euclidian distance method; and gene ontology, GoProfiles (goProfiles_1.20.0.tar.gz), (available at http://bioconductor.org/biocLite.R) with outputs plotted with gplots_2.11.0.tgz and user generated software. Human and mouse genes were cross referenced using the Human and Mouse Orthology (*HUGO Gene Nomenclature Committee*) file (HMD_HGNC_Accession.rpt) obtained from the Comparative Genome Databases (http://www.informatics.jax.org/orthology.shtml). The microarray data have been deposited in NCBI’s Gene Expression Omnibus [65] and are accessible through GEO Series accession number GSE48200 (http://www.ncbi.nlm.nih.gov/geo/query/acc.cgi?acc=GSE48200).

### Statistical analysis

Statistical significance was measured using the student t-test (Numbers, 09 v.2.0.4) using the parameters of paired or equal variance and two-tailed significance (unless otherwise indicated) with a probability of ≤ 0.05 considered as statistically significant. The Chi-squared test was performed using GraphPad http://graphpad.com/quickcalcs/contingency1.cfm. Values were calculated as means +/− standard deviation for sample size > 2 and +/− standard error for sample sizes of 2 (for some of the values as indicated).

### Genetic analysis of NS1

The amino acid sequences of NS1 from various IAV subtypes were retrieved from the NCBI’s Influenza Virus Resource; (http://www.ncbi.nlm.nih.gov/genomes/FLU/). The frequency of the amino acid in NS1 position 103 and 106 (in reference to the A/Hong Kong/1-5-MA21/1968 strain) was calculated using the Genenious Ver 6.0.5 analysis tool.

### Phylogenetic analysis

A total of 47 complete NS1 protein sequence of IAV, retrieved from the NCBI’s Influenza Virus Resource, were included for the representative phylogenetic tree construction (the full tree is shown in Additional file 1: Figure S2). Protein sequences were aligned using MUSCLE 3.7, with a number of iterations of 12. Maximum-likelihood analysis was performed with Geneious Ver 6.0.5, using the WAG substitution model using 1000 bootstrap replicates. GenBank accession numbers used for phylogenetic reconstruction can be found in supplementary information (Additional file 1: Table S2).

## Results

### 103L and 106I in the H5N1 NS1 gene each control virulence in mice

**Figure 1:**
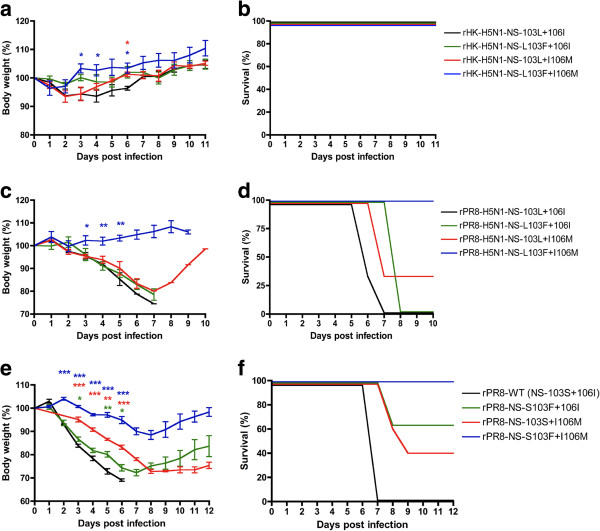
**Virulence is increased by each of the 103L and 106I NS1 mutations in the H5N1-NS gene on human and mouse adapted virus backbones as well as for 103S and 106I PR8 NS1 gene mutations in PR8 virus. a** and **b**. Groups of 3 CD-1 mice were infected intranasally with 5 × 10^6^ pfu of rHK viruses possessing wt (103L + 106I) or mutant H5N1 NS1 genes that differed due to the indicated mutations at positions 103 and 106. **c** and **d**. Groups of 3 CD-1 mice were infected intranasally with 1 × 10^4^ pfu with the different rPR8 viruses possessing wt (103L + 106I) or mutant H5N1 NS1 genes that differed due to the indicated mutations at positions 103 and 106. **e** and **f**, Groups of 5 BALB/c mice were infected intranasally with 1 × 10^4^ pfu with wt rPR8 or mutants that differed due to the indicated mutations at positions 103 and 106. The percent of body weight loss was calculated as the mice body weight loss was recorded daily throughout the whole course of the experiment. Values are shown as average +/− standard deviation (*p<0.05, **p<0.01, *** p<0.001; two-tailed student’s t-test).

To assess the virulence phenotypes associated with the individual 103L and 106I sites the H5N1 NS-wt genome segment that possesses the 103L and 106I mutations as its wt reference sequence or mutants with L103F or I106M mutations alone or in combination were rescued into a human and mouse-adapted backbones, A/HK/1/68 (H3N2) (HK) and A/PR/8/34 (H1N1) respectively. The relative virulence of these viruses on the HK and the PR8 backbones were assessed in groups of 3 and 7 Swiss-Webster (CD-1) mice following intranasal infection with 5.0 × 10^6^ pfu and 1.0 × 10^4^ pfu respectively. The rHK-H5N1-NS-wt (103L+106I) and the three mutants (rHK-H5N1-NS-L103**F**+106I, rHK-H5N1-NS-103L+I106**M** and rHK-H5N1-NS-L103**F**+I106**M**) did not result in mortality in CD-1 mice (all had LD_50_ >10^7.2^ pfu) (Figure 1b). Daily body weight monitoring showed the greatest loss for the rHK-H5N1-NS-wt (103L+106I) virus (7% at day 5 pi). Each of the 3 mutant caused significantly less body weight loss on comparison of days 3–6 for H5N1-NS mutants L103**F**+106I, 103L+I106**M** and L103**F**+I106**M** (P≤0.01, P≤0.05, and P≤0.001 by paired t-test respectively); and single time points showed significant differences for 103L+I106**M** and L103**F**+I106**M** (P≤0.05 by single sample t-test, n = 3) (Figure 1a). Thus each of the individual 103L and 106I mutations contributed to disease severity which was cumulative in combination.

Mouse infections with 1 × 10^4^ pfu of rPR8-H5N1-NS-wt (wt sequence 103L+106I) resulted in 100% mortality by 8 dpi with a 26 % average body weight loss (Figure 1c and d). Further infections of groups of mice with reduced dosages indicated an LD_50_ of 10^2.8^ pfu. The PR8 recombinants possessing the single mutations, H5N1-NS-L103**F**+106I and H5N1-NS-103L+I106**M**, had slightly reduced or delayed mortality (Figure 1d) associated with similar LD_50_ values of 10^3.0^ and 10^3.6^ pfu respectively, and body weight losses similar to H5N1 NS-wt (Figure 1c). The double mutant rPR8-H5N1-NS-L103**F**+I106**M** did not cause mortality, (LD_50_ of > 10^6.5^pfu) and induced significantly less body weight loss (maximum 4%) than H5N1 NS-wt (P ≤ 0.05 after day 3 by t-test) (Figure 1c). Therefore, each of the 103L and 106I mutations in the H5N1 NS gene was associated with enhanced disease to increase virulence (based on reduction in LD_50_ relative to F103 + M106) by >10^2.9^ (>800) and >10^3.5^ (>3,200) fold respectively, relative to >10^3.7^ (>5000) fold in combination when assessed on the mouse adapted A/PR/8/34 backbone. Thus each of the F103L and M106I mutations resulted in similar levels of increased virulence but this was dependent of the virulence of the genetic backbone with the greatest effects seen on a virulent virus backbone.

The properties of 103 and 106 mutations were also studied in the context of the A/PR/8/34 (H1N1) NS gene, that possesses 103S and 106I (considered as wt relative to the consensus sequence of 103F and 106M), on the same mouse adapted A/PR/8/34 backbone. The PR8-NS1 103S and 106I sites were mutated to S103**F**, I106**M** and S103**F**+I106**M**. The four rPR8 viruses (rPR8-NS-103S+106I (wt), rPR8-NS-S103**F**+106I, rPR8-NS-103S+I106**M**, rPR8-NS-S103**F**+I106**M**) were used to infect groups of 5 BALB/c mice intranasally with 1.0×10^4^ pfu each. The rPR8-NS-103S+106I (wt) resulted in 100% mortality and 31% body weight loss by day 6 pi i (Figure 1e and f), and possessed an LD_50_ of 10^3.5^ pfu. The single NS1 S103**F** and I106**M** mutants had small decreases in virulence and resulted in 40 % and 60% mortality; LD_50_ = 10^4.1^ and 10^3.9^ pfu respectively; (Figure 1e and f). However, both NS1 mutations S103F+I106M in combination were avirulent at this dose and resulted in 11% body weight loss by day 8 pi (Figure 1e-f), (LD_50_ >10^4.5^ pfu). The disease severity as indicated by body weight loss showed that each mutation was associated with significantly less disease than PR8-wt virus with the greatest reduction in body weight loss associated with the I106M and a smaller though significant effect seen for S103F mutations (that in combination reduced severity the most) (Figure 1e) (P values ≤ 0.05 – 0.001 by t test). Thus the analysis of the PR8 NS1 gene on its native backbone (PR8) further supports individual roles and cumulative effects for increasing virulence for mutations at positions 103 and 106 with a greater effect seen for the 106I than the 103S mutations.

### NS1 103L and 106I mutations in the H5N1-NS gene control the extent of alveolar infection in the lung

**Figure 2:**
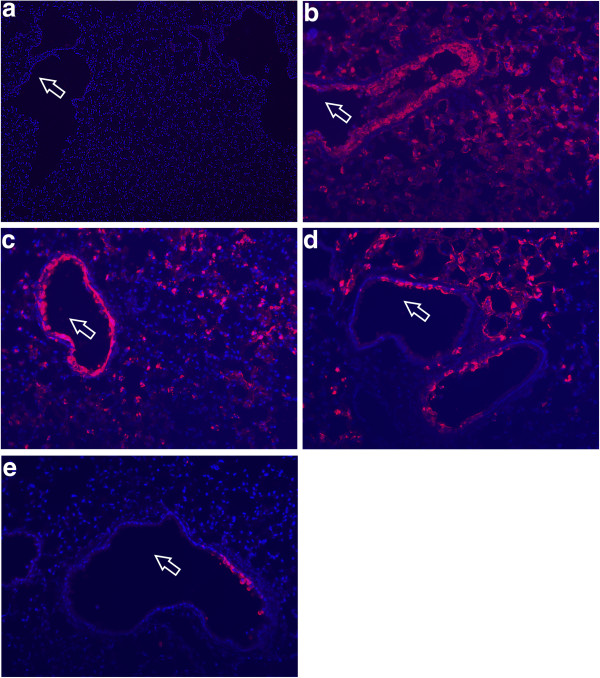
**103L, 106I and 103L+106I mutations in the H5N1-NS gene enhance viral tropism for alveolar tissue in mouse lungs.** CD-1 mice were infected intransally with 1 × 10^5^ pfu and lungs were collected 3 dpi. The lungs were inflated and fixed with 3.7% formaldehyde and frozen lung sections were stained with anti-PR8 antibody. The bronchioles are indicated by an arrow. **a**. Mock uninfected lungs **b**. rPR8-H5N1-NS-(L103 + I106) infected lungs **c**. rPR8-H5N1-NS-L103F + I106 infected lungs **d**. rPR8-H5N1-NS-L103 + I106M infected lungs **e**. rPR8-H5N1-NS-L103F+I106M infected lungs.

To further characterize the properties of the 103L and 106I mutations in the H5N1-NS gene on the rPR8 backbone the extent of mouse lung infection and tissue tropism was determined by immunofluorescent staining of frozen lung sections at 3 days post intranasal infection with 1.0 × 10^5^ pfu of each mutant virus. The lungs infected with the rPR8-H5N1-NS-wt (103L+106I) virus showed extensive virus infection and spread that encompassed the bronchi as well as the alveolar regions (Figure 2b). When individually mutated back to consensus, the L103F and I106M mutants showed a decrease in the extent of alveolar tissue infection (Figure 2b-d). The rPR8-H5N1-NS-L103**F**+I106**M** mutant was severely restricted in lung infection, showing small regions of bronchiolar with rare foci of alveolar infection (Figure 2e). Thus the enhanced virulence due to each of the 103L and 106I mutations was associated with increased alveolar tissue infection.

### 103L and 106I in H5N1-NS increase viral replication in mouse lungs

**Figure 3:**
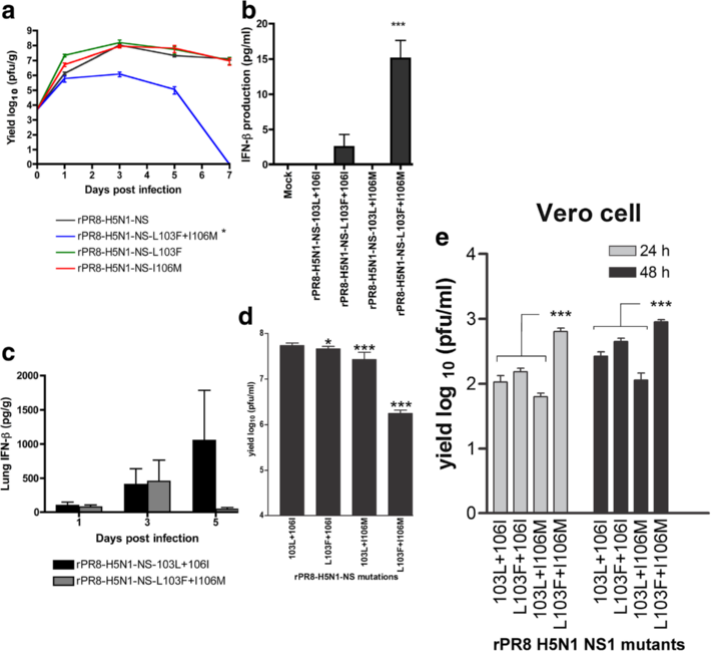
**H5N1-NS gene 103L, 106I and 103L+106I mutations enhance viral replication in mouse lungs and increase IFN-β antagonism in mouse cells. a**. Groups of 12 CD-1 mice were infected intransally with 5x10^3^ pfu of the rPR8-H5N1-NS and the three mutants (rPR8-H5N1-NS-L103F, rPR8-H5N1-NS-I106M and rPR8-H5N1-NS-L103F+I106M). Lung homogenates were collected at days 1, 3, 5 and 7 pi (n = 3) and viral titers were assessed by plaque assay. The L103F+I106M mutant generated 100 fold less virus than the others (*p<0.05; by paired two-tailed t-test). **b**. IFN-β induction in mouse M1 cells. Monolayers of M1 cells in 35 mm dishes were infected at MOI = 2 and incubated for 24 h before collecting supernatants for quantification of IFN-β by ELISA (n = 3 biological replicates with n = 2 technical replicates). The values for rPR8-H5N1-NS-wt and NS1 L103F + I106M were first reported by [13] and are included here as controls. Values are shown as average +/− standard deviation with significant difference indicated for NS1 L103F + I106M relate to each of the other mutants (*** p<0.001; two-tailed t-test). **c**. IFN-β induction in mouse lungs. Groups of 12 CD-1 mice were infected intransally with 5x10^3^ pfu of the rPR8-H5N1-NS and the rPR8-H5N1-NS-L103F+I106M. The levels of IFN-β lung homogenates were determined by ELISA at days 1 and 3 (n =3), and 5 (n =6) pi. **d**. Infectious yield associated with IFN-β induction in mouse M1 cells shown in panel B were quantified for infectious yield by plaque assay. The yield of the L103F+I106M mutant was significantly less than the other 3 viruses (*** p<0.001; two-tailed t-test). **e**. Infectious yield in monkey Vero cells were quantified for infectious yield by plaque assay. The yield of the L103F+I106M mutant was significantly greater than each of the other 3 viruses (*** p<0.001; two-tailed student’s t-test).

Given that the H5N1-wt NS gene possessing 103L and 106I residues increased virulence and alveolar tropism we then assessed virus yield in lung tissues. CD-1 mice were infected intranasally with 5 × 10^3^ pfu of the rPR8-H5N1-NS-wt (103L+106I) virus as well as the three mutants (L103**F**, I106**M** and L103**F** + I106**M**). Lungs from 3 mice were collected at each of days 1, 3, 5 and 7 pi and the lung titers were determined by plaque assay. The rPR8-H5N1-NS-wt virus grew to a high titer in CD-1 mouse lungs with a peak yield of 1.05 × 10^8^ pfu/g at day 3 pi (Figure 3a). When each of the 103L and 106I sites in the H5N1-NS gene was individually mutated back to consensus, virus replication of either of the L103**F** and I106**M** mutants was not significantly different from the wt (103L+106I) virus (P ≥ 0.05 by single sample and paired t-test) (Figure 3a). However when both sites were mutated in combination, the H5N1-NS-L103**F**+I106**M** virus possessed severely decreased viral replication (>100 fold less at day 5 pi) (P ≤ 0.01 by paired t-test from days 1–5, n = 9) compared to the rPR8-H5N1-NS-103L+106I, and the virus was cleared from the lungs by day 7 pi (Figure 3a). Therefore the presence of either one of the 103L and 106I mutations in the H5N1 NS gene resulted in a large increase in virus replication and a delay in virus clearance in lungs.

We had shown earlier that the rPR8-H5N1-NS-103L+106I virus suppressed IFN-β production to undetectable levels in M1 cells infected at an MOI = 2 at 24 hpi [13] whereas the L103**F**+I106**M** mutant generated low but measurable levels (15 ng/ml). Measurement of the IFN-β induced by the 2 single mutants L103**F**+106I and 103L+ I106**M** showed that both were able to significantly suppress IFN production relative to the consensus virus that was undetectable in the case of 103L+ I106**M** (Figure 3b).

We then assessed IFN-β production *in vivo* for the rPR8-H5N1-NS-103L+106I (wt) and rPR8-H5N1-NS-L103F+I106M lung homogenates. Each of these viruses produced similar lung IFN-β levels at days 1 and 3 pi (P ≥ 0.05, equal variance t-test) (Figure 3c). At day 5 pi the 103L+106I mutant possessed much higher average lung IFN-β levels than the L103F+I106M mutant (1054 pg/g versus 95 pg/g respectively) although not to a statistically significant level (which did not become significant after increasing the number of mice to 6 per treatment (P ≥ 0.05, t-test (n = 6)) (Figure 3c). Thus the *in vivo* IFN-β production for the 103L + 106I mutant contradicted the *in vitro* findings which showed reduced IFN induction in M1 cells (Figure 3b).

We then measured the virus yield in the M1 cell supernatants analyzed for IFN-β induction in Figure 3b and showed that each of the L103L and 106I mutants demonstrated increased virus replication with the greatest effect seen for the H5N1-NS1wt virus possessing both 103L+ 106I mutations (Figure 3d) consistent with increased replication associated with increased ability to antagonize IFN production, which paralleled the increased replication seen for these mutants in mouse lungs (Figure 3a).

To assess the function of these mutations independent of the IFN response we measured virus yields in Vero cells (that lack IFN genes) infected at moi = 0.01 with plaque assay at 24 and 48 hpi for this set of NS1 mutants. Although Vero cells are poorly permissive for PR8 replication [66], virus replication is independent of IFN production. Each of 103L and 106I mutations significantly decreased virus yield with an intermediate effect for the double 103L + 106I mutant indicating that these mutation provide an IFN independent loss of replicative function in Vero cells. This is not inconsistent with the enhanced IFN antagonism mediated gain-of-function seen for these mutations in A/HK/1/68 (H3N2) viruses in mice [15].

### H5N1-NS residues 103L and 106I increase NS1 cytoplasmic accumulation

**Figure 4:**
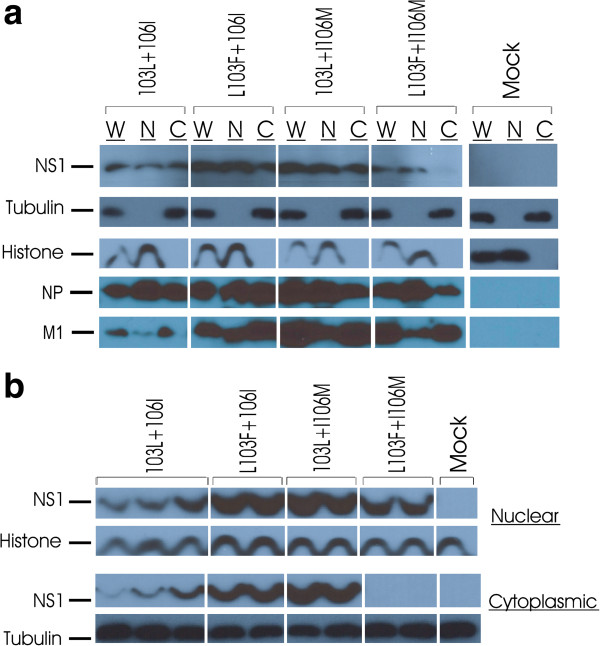
**The 103L and 106I residues in the H5N1-NS gene are associated with increased cytoplasmic localization of NS1 protein in mouse epithelial cells.** Monolayers of mouse epithelial cells were infected with rPR8-H5N1-NS-103L+106I (n=3) and the three mutants rPR8-NS-L103F+106I, rPR8-NS-103L+I106M and rPR8-NS-L103F+I106M (n=2) at MOI=2 and compared to mock uninfected cells. NS1, NP and M1 proteins localization was detected by western blots using rabbit protein specific antibodies. Rabbit anti-tubulin and mouse anti-histone antibodies were used as controls for cytoplasmic and nuclear fractions respectively. **a**. The nuclear and cytoplasmic accumulation of NS1 protein is shown for one analysis. The whole cell lysate is designated as “W”, nuclear fraction as “N” and the cytoplsmic fraction as “C”. **b**. Western blots showing the H5N1-NS1 protein nuclear and cytoplasmic fractions in parallel for 2–3 replicates of infected M1 cells.

The PR8 NS1 F103S and M106I mutations have been reported to function to increase cytoplasmic localization in MDCK cells [47] and we have seen similar results for the F103L and M106I mutations in the HK1 NS1 gene that resulted in increased cytoplasmic accumulation in mouse M1 cells (N.E. Forbes et al. submitted). To determine whether the replicative gain-of-function of the H5N1-NS1 103L+106I mutations (Figure 3d) involves altered cytoplasmic accumulation the presence of NS1 protein in nuclear and cytoplasmic fractions (along with NP and M1 proteins) was assessed in mouse epithelial M1 cells *in vitro*. Monolayers of M1 cells were infected with the rPR8-H5N1-NS-wt (103L+106I) virus and the three mutants (L103**F**+106I, 103L+I106**M** and L103**F**+I106**M**) for sixteen hours before whole cell lysate was collected and the nuclear as well as the cytoplasmic fractions were separated. Tubulin and histone H3 were used as cytoplasmic and nuclear markers respectively. The NS1 protein accumulation observed by western blots show that the 103L and 106I NS1 residues both functioned to increase cytoplasm abundance for the viruses possessing the L103F+106I, 103L+I106M and 103L+106I NS1 residues relative to the L103F+ I106M virus (Figure 4a). Western blot of the NP protein showed it was primarily nuclear with greater accumulations for the 3 viruses containing 103L and 106I mutations (relative to L103F+I106M) whereas the M1 protein expression was primarily cytoplasmic where the 103L+106I variant was lowest relative to the other viruses (Figure 4a) indicating mutation specific patterns of gene expression. Duplicate or triplicate experiments were then analyzed to confirm the initial NS1 accumulation observations (Figure 4b). Interestingly, the NS1 protein cytoplasmic accumulation was at the limit of detection for the L103F+I106M virus in mouse M1 cells, which correlated with a significantly restricted ability to replicate in vitro (Figure 3d). The single mutants L103F+106I and 103L+I106M also enhanced the total and nuclear NS1 protein expression 16 hpi compared to the 103L+106I infected cells (relative to 103F+106M) indicating that the 2 mutations in combination are less able to support increased general gene expression (especially for the M1 protein) suggesting that the gain-of-function due to the NS1 mutations involves changed NS1 protein function and or location rather than enhanced NS1 protein synthesis (Figure 4b).

### 103L and 106I enhance human IFN-β transcription inhibition but differ in binding to cleavage and polyadenylation specificity factor 30 F2F3 domain (CPSF30-F2F3)

**Figure 5:**
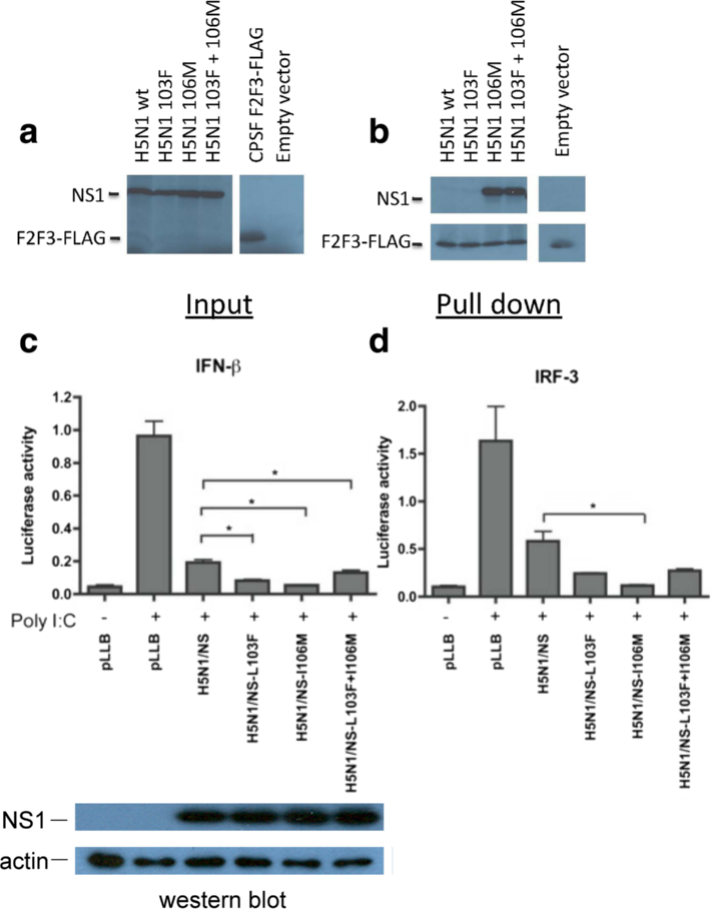
**The 103L and 106I residues in the H5N1-NS gene antagonize poly I:C induced IFN-β and IRF-3 promoter transcriptional activation but differ in CPSF-30 F2F3 binding. a**. H5N1-NS1 proteins (wt and mutants) as well as CPSF30-F2F3-FLAG were expressed by coupled *in vitro* transcription and translation of expression plasmids or empty vector in the presence of ^35^S labeled methionine and cysteine with detection by autoradiography. Radiolabeled NS1 proteins or empty vector were mixed with radiolabeled CPSF30- F2F3-FLAG and subject to pull-down using α-FLAG M2 antibody and detected by SDS-PAGE and autoradiography. **b**. Representative pull-down of 2 independent replicates showing that the I106 mutation abrogated binding but L103 maintained binding. **c**. IFN-β promoter activation was measured in human 293-T cells in triplicate biological replicates for the H5N1-NS-wt and the three NS mutant (L103F, I106M and L103F+I106M) expression plasmids using a dual-luciferase reporter assay. Each of the NS expression plasmids along with the firefly luciferase p125 under the control of the human IFN-β promoter as well as the renilla luciferase control pRL-SV40 reporter plasmids were transfected in 293T cells. Sixteen hours following transfection, IFN-β promoter activation was stimulated by poly I:C transfection. The luciferase activities were measured following a 24h incubation period and are shown as the ratio relative to the internal renilla luciferase control. **d**. The same experiment was repeated using the firefly luviferase reporter plasmid under the control of the human IRF-3 promoter. Data represent the means of n = 3 values ± SD (*p<0.05, **p<0.01, *** p<0.001; two-tailed student’s t-test) of firefly luciferase activity relative to renilla.

The rPR8-H5N1-NS mutations 103L and 106I decreased *in vitro* IFN-β production in mouse cells that correlated with enhance infection and virulence *in vivo* (Figure 3). To further understand the ability of the 103L+106I NS1 sites to affect host dependent aspects of IFN-β production, IFN-β inhibition was examined in human cells by assessing the effect on IFN-β promoter activation and by assessing CPSF-30 F2F3 binding ability. Transfection with H5N1 NS1-wt and mutant pLLB plasmids were performed in 293T cells co-transfected with firefly luciferase reporter plasmids under the control of human IFN-β (p125) or IRF-3 (p55C1B) promoters plus an internal *Renilla* luciferase reference standard; followed by IFN-β induction with poly I:C dsRNA at 16 h post-transfection. The H5N1-NS-wt gene possessing the 103L+106I residues inhibited IFN-β transcription compared to the empty control pLLB vector however the presence of the consensus amino acids 103F, 106M and 103F+106M were further reduced in IFN-β promoter activation by significant amounts (P ≤ 0.05 by t-test) (Figure 5c). Transcription inhibition of the IRF-3 promoter showed a similar pattern (P ≤ 0.05 by t-test) (Figure 5d). The greatest IFN-β and IRF-3 transcription inhibition was observed for the NS1 gene possessing the 103L+106M residues.

The 103 and 106 sites map to CPSF-30 binding domains [67] therefore we assessed NS1 binding to the FLAG-F2F3 fragment of CPSF30 in the context of H5N1-NS gene mutants. Equal expression of each NS1 protein as well as FLAG-F2F3 was verified by autoradiography (Figure 5a). In the FLAG-F2F3 pull down, the H5N1 NS1 possessing either L+I or F+I at positions 103 and 106 showed undetectable FLAG-F2F3 binding (Figure 5b), however, the mutation I106M in the H5N1-NS gene restored binding to FLAG-F2F3 (for 103L+I106M and L103F+I106M).

Thus we observed that the combination of NS1 103L+I106M and L103F+I106M mutations both bound to CPSF30-F2F3 but 103L+I106M differed by enhancing IFN-β and IRF-3 inhibition of transcription in humans (Figure 5). However, the H5N1-NS gene (L+I) did not bind CPSF-F2F3 (Figure 5b) and possessed the lowest ability to inhibit human IFN-β-promoter activation among the mutants (Figure 5a and b) but was capable of inhibiting IFN-β induction in mouse cells to undetectable levels (Figure 3) indicating that IFN antagonism in human cells differs for this combination of mutations.

### 103L and 106I H5N1-NS residues increase binding to human RIG-I domains

A bacterial reverse two-hybrid system has been previously used to determine the strength of the protein-protein interactions between A/WSN/1933 NS1 and the CARD, Helicase and RD domains of human RIG-I (NS1 was found to bind RD and CARD but not the helicase domain of RIG-I [35]). Here, we used the same system to determine the affinity of NS1 from different IAV strains for the domains of human RIG-I. A separate RTHS was constructed for each protein pair; the targeted proteins are expressed from a chromosomally-integrated, IPTG-inducible cassette as N-terminal fusions with 434 or P22 repressor domains (Additional file 1: Figure S1). The interaction of the IPTG-induced proteins reconstitutes a functional 434/P22 chimeric repressor that prevents transcription of reporter genes downstream. If the target proteins do not interact, a functional repressor will not form, and the host cells will express the reporter genes independent of IPTG levels.

**Figure 6:**
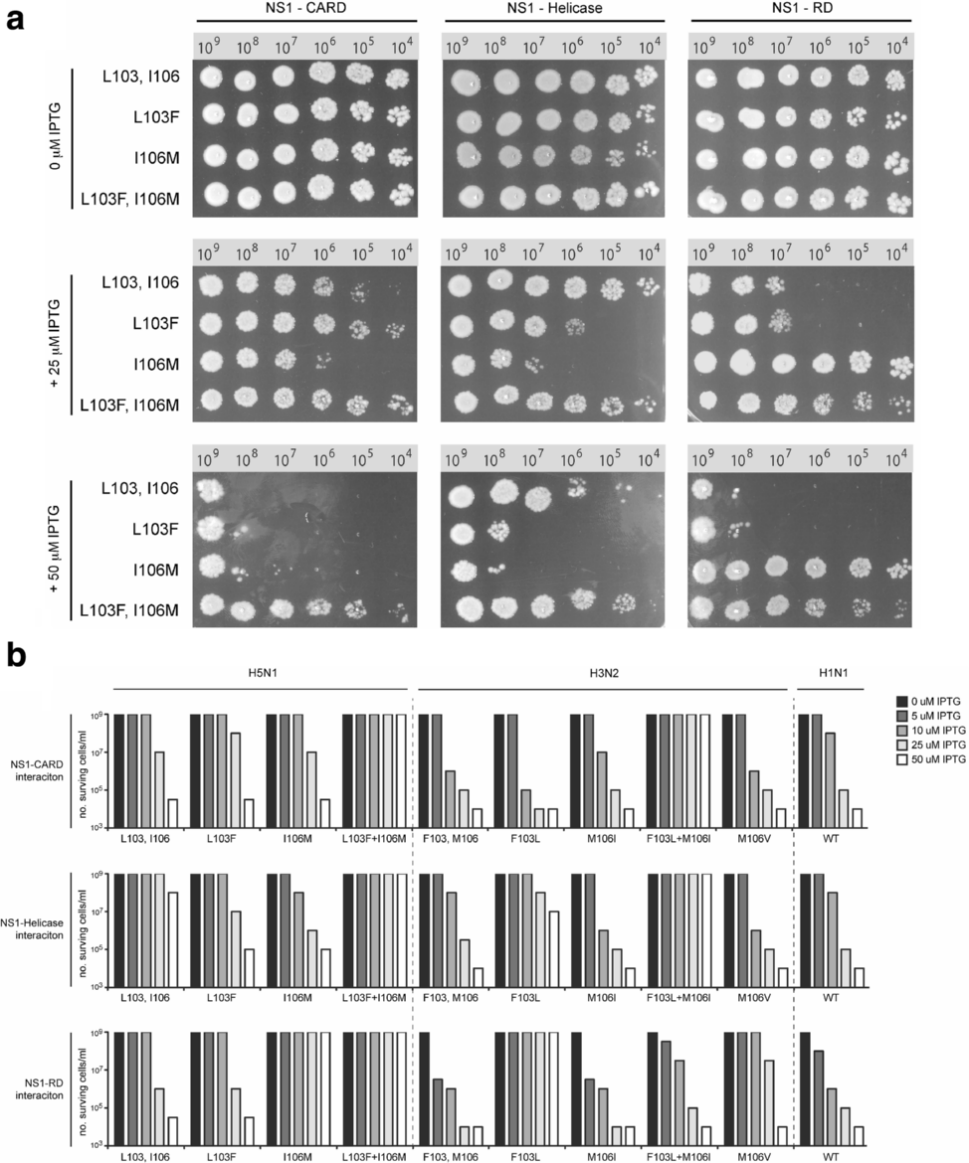
**The H5N1 NS1 L103 and I106 mutations affect binding of NS1 to human RIG-I CARD, helicase and RD domains in the bacterial reverse 2-hybrid system. a**. Drop spotting of the H5N1 influenza NS1 RTHS strains on selective media. Serial dilutions (2.5 μL of each sample containing ~10^n^ cells/mL is indicated) of the NS1 RTHS strains (and controls), were drop spotted onto selective media containing 0, 25 or 50 μM IPTG. In the absence of IPTG, there is no expression of the targeted fusion proteins; all strains therefore grow normally (top row of plates). Upon addition of IPTG (2nd and 3rd row of plates), the functional 434.P22 repressor will be reconstituted in those RTHS that contain interacting proteins (Additional file 1: Figure S1), leading to cell death. The strains containing proteins that do not interact (e.g. L103F, I106M) continue to grow normally in the presence of IPTG. **b**. Dose response for IPTG induction of proteins for RIGI domain binding of H5N1 and H3N2 NS1 L103 and I106 mutants as well as H1N1 and H3N2 wt NS1 proteins. RTHS constructs that possessed each of the wt H5N1 (A/HK/156/1997), H3N2 (A/HK/1/68) and H1N1 (/Brevig Mission/1/1918) or indicated 103 and 106 mutant for the H5N1 and H3N2 viruses were tested for survival under selective medium on induction with 0, 5, 10, 25 and 50 μM IPTG. The number of surviving bacterial colonies were determined by serial dilution as shown in panel A and plotted as bar graphs. NS1 proteins that do not bind the indicated RIG-I domain baits (CARD, helicase and RD) were not reduced in survival relative to the non-induced controls (0 μM IPTG).

RTHS were built for the interaction of NS1 from the H5N1 (A/HK/156/1997), H3N2 (A/HK/1/68) and H1N1 (A/Brevig Mission/1/1918) strains of influenza, with the CARD, helicase and RD domains of RIG-I. In addition to the wild-type NS1, residue 103 and 106 H5N1 and H3N2 mutants detailed above were also assessed by this method, with a RTHS built for each mutant. We quantified the targeted protein-protein interaction by drop spotting ten-fold serial dilutions of the above NS1 RTHS strains onto selective media containing 3-amino-1, 2, 4-triazole (2.5 mM) and kanamycin (25 μg/mL) in the presence of increasing IPTG levels (Figure 6a). The H5N1 data showed that the wt NS1 (103L+106I) protein interacts strongly with CARD and RD domains of RIG-I, but not with helicase. The L103**F**+106I mutant interacted strongly with all three RIG-I domains, whereas an I106**M** mutation (103L+I106**M**) eliminates the interaction of NS1 with RD but restored helicase binding to the helicase domain. Interestingly, the L103**F**+I106**M** double mutant, with the NS1 consensus sequence, was unable to interact with any of the RIG-I domains. These data indicated that both the F103L and M106I mutations modulate RIG-I binding in the H5N1 NS1 gene.

To further explore the role of point mutations in NS1’s interaction with the domains of RIG-I, we repeated this experiment at 0, 5, 10, 25 and 50 μM IPTG, for the H5N1, H3N2 and H1N1 strains. The extent of survival of each RTHS *E*. *coli* strain was quantified via the dilution level at which colonies survived and grew on selective media at various IPTG levels (Figure 6b). The RTHS titration data confirmed the above observations for the H5N1 strain (that I106M mutant does not bind RD and L103F + I106M mutant does not bind any of the RIG-I domains). The wt H1N1 and H3N2 NS1 proteins both bind strongly to all three domains of RIG-I suggesting that the lack of helicase binding of WSN NS1 may be due to laboratory adaptation. We also tested each the 103L and M106I mutations in the H3N2 NS1 gene where the M106I mutation did not affect binding to RIG-I domains but the F103L mutation eliminated binding to helicase and RD, which were the same binding patterns as seen for the H5N1 NS1 genes with these amino acid combinations (Figure 6). The H3N2 F103L + M106I double mutant demonstrated binding defects to lose the ability to bind CARD and helicase, and showed weaker interaction with RD than wt H3N2 NS1. Interestingly, a M106V mutation, which was also previously described to be selected on mouse adaptation [15], did not affect the interaction with CARD or helicase, but significantly weakened the interaction with RD.

These data demonstrated that RIG-I domain binding was positively affected by mutation at positions 103 and 106 in the avian H5N1 NS1 gene that were also subject to epistatic affects due to NS1 gene differences between the human HK and avian-like H5N1 NS1 proteins. Thus IFN antagonism properties may also be affected by differences in ability to bind the cytoplasmic RNA sensor RIG-I.

### The 103L and 106I mutations in the human A/HK/1/1968(H3N2) NS1 gene show differential and host dependent effects on host gene transcription in human and mouse cells

**Figure 7:**
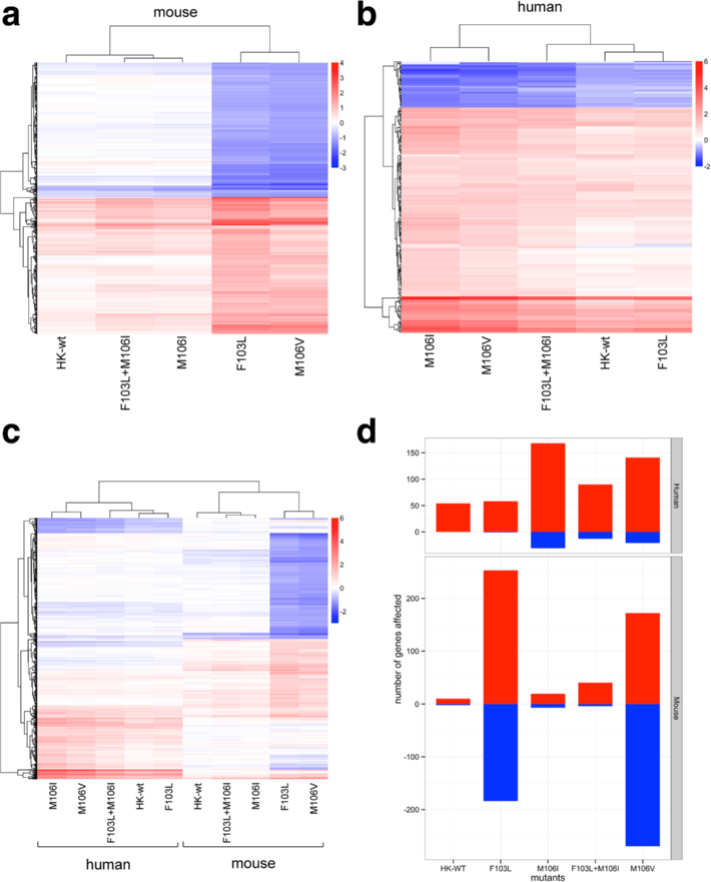
**Host gene expression is differentially affected by 103L and 106I mutation in human and mouse cells.** Mouse M1 cells and human A459 cells were wither mock infected with PBS of infected with rHK1-wt, or NS1 F103L, M106I, M106V and F103L + M106I mutants at MOI = 2 in triplicate with extraction of total RNA at 8 hpi. Genes that were significantly increased or decreased 2 fold (≥ 2^1^ or ≤ 2^-1^) by Affymetrix microarray analysis relative to mock infected cells for any individual virus were analyzed among the viruses. Host genes are represented in heat maps with red and blue according to with the indicated scales for the log 2 values **(**panels **a**, **b** and **c)**. **a)** Effect of virus infection on gene regulation in mouse M1 cells showing hierarchical cluster analysis of dys regulated genes as well as hierarchical clustering among viruses. **b)** Effect of virus infection on gene regulation in human A549 cells showing hierarchical clustering of genes and viruses as represented in panel a. Relative to HK-wt the 242 dysregulated genes were significantly upregulated for M106I, M106V and F103L + M106I (** p ≤ 0.01, *** p ≤ 0.001 by t-test) but not F103L (not significantly different (nsd)). **c)** Transcriptional abundance analysis of genes that are common between human and mouse cells were analyzed as described in panel a. **d)** Bar graph of up (red bars) and down (blue bars) regulated gene transcripts detected by microarray analysis relative to mock infected M1 and A459 cells infected with the indicated viruses. The number of significantly up and down regulated genes relative to mock infected A549 cells was significantly different for M106I, M106V and F103L + M106I (*p ≤ 0.05 by Chi-squared test), versus HK-wt (reference, r) but not F103L that was not significantly different.

Because RIG-I and CPSF binding are associated with host gene regulation we performed genome-wide microarray analysis of mRNA abundance in human and mouse cells relative to mock-infected control cells. Microarray analysis was performed using the mouse Affymetrix Genechip Mouse Exon 1.0 ST Array on RNA extracted from mouse M1 cells 8 hours following infection with HK-wt and NS1 mutants at a MOI of 2 that were compared to a parallel analysis of infected human A549 cells that were analyzed using the human Affymetrix Genechip Mouse Exon 1.0 ST Array. Three independent infections were analyzed in parallel for each of the infected cell types with comparisons of genes that were significantly up or down regulated by ≥ 2 fold at the p ≤ 0.05 level by ANOVA (n=3) relative to mock infected M1 and A549 cells. The analysis included HK-wt virus and mutants possessing F103L, M106I, and F103L + M106I in addition to anther adaptive mutant M106V [15]. Analysis of the transcription profile of HK-wt or mutant infected mouse M1 cells relative to mock infected (PBS) control cells showed 573 genes that were greater than 2 fold up or down regulated in at least one of the mutants (Figure 7a and d) where the F103L and M106V induced larger effects on host gene expression (253 and 172 up-regulated versus 184 and 269 down-regulated genes respectively) than the wt (Figure 7d) that had a lesser effect on host gene expression (10 up-regulated and 0 down regulated genes) and clustered with the M106I and F103L+M106I double mutant (19 and 40 versus 7 and 4 down-regulated respectively) (Figure 7a and d). Thus the effect of the M106I mutant was dominant when expressed in combination with the L103 mutant in infected mouse cells. These gene expression patterns were independent of CPSF binding, since all mutants had lost CPSF binding except F103L that maintained detectable binding in the HK NS1 protein (2% of HK-wt) [15] and unpublished data for the double mutant).

**Figure 8:**
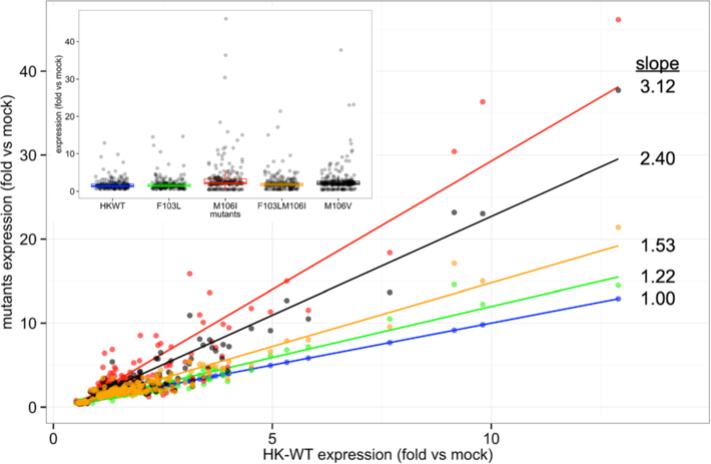
**Linear plot of relative gene expression of host genes between NS1 HK**-**wt and mutants in human A459 cells.** The host gene expression relative to HK-wt was plotted with the line of best fit for dysregulated genes in human A459 cells shown in Figure 7a. The slope of each line is indicated relative to the HK-wt line with slope = 1. Insert. A box plot of dysregulated gens from Figure 7a shown relative to the gene expression of HK-wt relative to mock infected M1 cells.

In the human cells the effects on host gene expression differed from the mouse by differentially affecting fewer genes, that in total affected the expression of 242 genes (Figure 7b). The HK-wt and F103L mutant had the least effect on host gene expression with 54 and 58 up-regulated versus 0 and 1 down-regulated genes respectively (Figure 7d). These genes tended to cluster together and were further linked with the F103L+M106I double mutant (90 up-regulated versus 13 down-regulated genes (Figure 7b and d)). In contrast the M106I and M106V mutants had the greatest effect on A549 host gene expression (168 and 141 up-regulated versus 31 and 21 down-regulated genes respectively) and clustered together. The HK-wt and F103L mutant viruses that both possessed CPSF30 binding (although reduced for F103L in the HK NS1 [15]) had significantly less effect on gene expression than the viruses that had lost CPSF30 binding (M106I, M106I + F103L, and M106V) by t test (p ≤ 0.001) (Figure 7b). This was also reflected in the number of statistically significant dysregulated genes (P ≤ 0.05 by Chi-squared test) (Figure 7d). We next assessed whether genes that were unregulated due to NS1 mutations were affected in consistent patterns relative to their expression in HK-wt infected cells. We compared the gene expression of dysregulated genes for each of the mutants to their expression in HK-wt infected cells (Figure 8). The dysregulated genes had a linear relationship of relative expression to the HK-wt (Figure 8) where the increased slope indicated the relative factor for increase in human gene expression that was effected by each mutant (line of best fit slopes of 3.1, 2.4, 1.5 and 1.2 for the M106I, M106V, F103L + M106I and F103L relative to HK-wt slope of 1) also shown with a box plot of expression levels for each mutant (Figure 8 insert). Thus mutations that increased dysregulation of host genes had a uniform effect to modulate expression by a consistent factor.

The relative patterns of gene expression were consistent with reduced host gene expression due to inhibition of CPSF30 mediated processing in human cells due to NS1 protein binding (Figure 7b) but this pattern was not observed in mouse cells (Figure 7a and d). The plot of gene expression for individual genes relative to HK-wt in mouse cells showed a similar pattern of expression for both M106I possessing mutants but an increased pattern of expression for F103L and M106V (Additional file 1: Figure S3).

We also analyzed the mouse and human data together to determine if gene regulation patterns were shared between the two hosts and found 656 shared dysregulated genes between human and mouse (Figure 7c) with 34 and 92 genes that were unique to the mouse and human respectively. In general we observed a large host-dependent effect where genes that were differentially up or down regulated in one host (red or blue respectively) were not differentially up or down regulated in the other host (Figure 7c).

#### Characterization of genetic variation of residues 103 and 106 among NS1 proteins in nature

**Figure 9:**
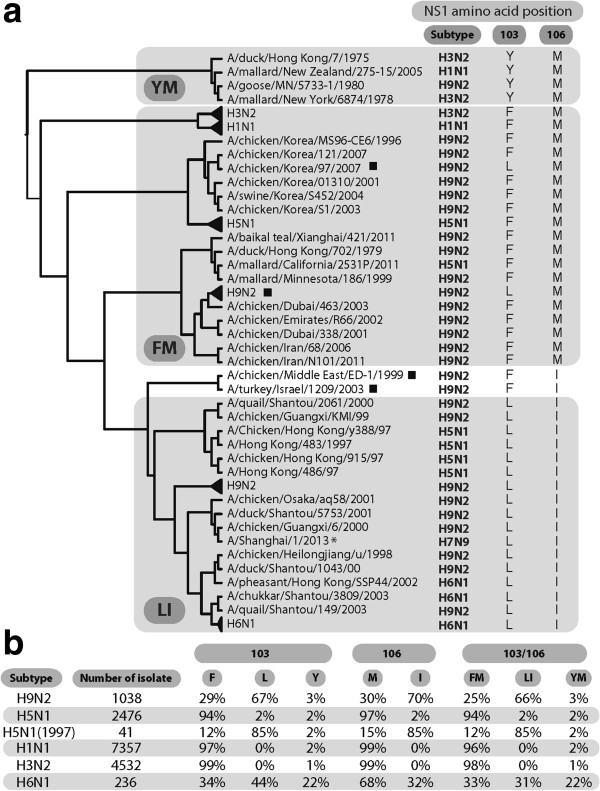
**Analysis of NS1 protein 103 and 106 residue mutation in natural human and animal isolates. a**. Phylogenetic trees based on the NS1 protein sequence of influenza A viruses isolated from subtypes H1N1, H3N2, H5N1, H6N1and H9N2. Know subtypes and amino acid at position 103 and 106 are indicated. Different 103/106 residue motifs (FM, YM, LI) are represented by a grey square. Full trees and a list of GenBank accession numbers of the viruses used are provided in supplementary information. Independent selection of F103L occurred in the FM lineage and are indicated with a square (■) and independent selection of M106I is shown for 2 H9N2 isolates representative of a Middle Eastern lineage. **b**. Variability found at position 103 and 106 in different IAV subtypes. H1N1, H3N2, H5N1, H6N1 and H9N2 viruses were analyzed for the frequency of different amino acid compositions at position 103, 106 and 103/106.

The prevalence of amino acid residues at position 103 and 106 was characterized for IAVes in the NCBI Influenza Virus Resources database to show that the NS1 protein from avian IAVs isolated before 1980 were F/M that were also characteristic of animal isolates or Y/M that was characteristic of NS1 allele B which has been maintained in a restricted subset of avian lineages. Avian IAVs isolated after 1990, in the H1N1, H5N1 and H3N2 lineage, predominately contain the F/M residues at position 103/106 (Figure 9), and similar observations can be made for most other subtypes: H8N4, H7N7, H6N8, H6N6, H6N5, H6N2, H5N3, H4N6, H4N2, H3N8, H3N6, H3N2, H2N9, H2N3, H2N2, H1N2, H1N1, H13N6, H11N9, H10N7 (data not shown). In contrast, in post-1990 H9N2 subtype viruses, the predominant residue at position 103/106 is L/I found in 66% of total isolates, therefore demonstrating positive selection of these residues in poultry for this subtype (Figure 9). The H6N1 lineage has also selected 103L and 106I mutations that represent 31% of total H6N1 isolates in the NCBI database (Figure 9). Although the H5N1 isolates are predominately F/M, most of the 1997 strains are L/I, due to reassortment with H9N2 viruses as also recently observed for the emergent H7N9 strains [2], in addition to the repeated independent selection of L/I residue in some H5N1 and H9N2 viruses (Figure 9). The first human H6N1 isolate, A/Taiwan/2/2013 (H6N1) possesses the F103L mutation (GISAID accession no. EPI_ISL_143275). This F103L mutation has been selected independently of the H9N2 lineage in the Taiwanese H6N1 sublineage (data not shown) which further links this mutation with adaptive evolution and avian to human host switching.

## Discussion

We had previously shown that the F103L+M106I mutations in H5N1-NS1 conferred high virulence on the PR8 backbone. Here we show that each of the F103L and M106I mutations in the H5N1 NS1 gene are gain-of-function mutations that increase IFN antagonism to overcome host resistance in the novel mouse host by increasing cytoplasmic NS1 expression with possible roles in altering RIG-I and CPSF30 host factor binding.

### Mutations at F103L and M106I enhance lung infection, replication, and virulence

Using reverse genetics and site directed mutagenesis we show that each of the F103L and M106I mutations increased virulence to approximately equivalent levels, (>800 and >3,200 fold respectively, and >5,000 fold in combination; on the basis of LD_50_’s of 10^3.6^, 10^3.0^, 10^2.8^ pfu respectively relative to the 103F + 106M mutant of >10^6.5^ pfu)) (Figure 1). This indicated that the L103 and 106I mutations both additively increased virulence in combination, with the greatest effect seen for the M106I mutation (4 fold more virulent than F103L). Each of the F103L, and M106I as well as the double F103L + M106I mutants resulted in enhanced infection of alveoli that was associated with >100 fold increased and prolonged viral replication relative to the consensus sequence 103F+106M virus that was attenuated and restricted to the bronchiolar infection of mouse lungs (LD_50_ >10^6.5^ pfu). This indicated that each of the F103L and M106I mutations overcame barriers to lung alveolar infection but their combined effects did not further increase virus yield *in vivo* and thus were both contributing to increased cytoplasmic accumulation, and possibly other functions, to enhance replication in the mouse. This is consistent with earlier studies on the human HK virus backbone that showed that the 106I mutation caused the largest loss in body weight (20%), among 8 single nucleotide adaptive mutants, and the F103L mutant induced a lesser weight loss of 10% [15].

### Cytoplasmic accumulation

Each of the 103L and 106I mutations increased cytoplasmic accumulation of the H5N1 NS1 protein that would be expected to increase NS1 cytoplasmic functioning. Both of these mutations have been shown to enhance cytoplasmic accumulation when introduced into the A/HK/1/68 (H3N2) backbone, (Forbes et al. submitted). Incorporation of 103L or 106I mutation may activate or enhance a secondary nuclear export function, in addition to the originally indentified NES at position 137-148 [68], to enhance interactions with exportin molecules or other parts of the nuclear pore complex [69,70]. Further support for this conclusion is that the 103S and 106I NS1 mutations in PR8 (H1N1) and A/Sydney/5/97(H3N2) control cytoplasmic versus nuclear localization in transfected MDCK cells [47]. The M106I mutation has also been shown to restore a defect in cytoplasmic localization and IFN antagonism of the 2003 lineage avian H5N1 NS1 gene in human A549 cells [71]. Mutations that result in increased binding to nuclear or cytoplasmic factors may also affect cellular trafficking by tethering NS1 protein to host proteins in specific locations such as cytoplasmic RIG-I and nuclear CPSF30 as shown for other proteins [69,72]. The biochemical basis for increased cytoplasmic localization could involve reduced binding to nuclear localized CPSF30; however the CPSF30 binding mutant 103F also trafficked to the cytoplasm arguing against CPSF30 binding playing a major role in nuclear sequestration of NS1 protein (Figure 4). But the fact that H5N1 NS1 103L mutant binds CPSF may account for its greater ability to inhibit expression from the IFN promoter, relative to the 106I mutant that did not bind CPSF.

### Effects of F103L and M106I on host gene transcription

The F103L and M106I mutations differed in their effects on host gene transcription where in mouse cells the 103L induced a large host gene regulation phenotype versus 106I that had a very small effect on host gene regulation that was dominant in combination in the double mutant. The numbers of deregulated host genes at 8 hpi for HK-wt (H3N2) in M1 cells were comparable to that published for mouse lungs infected with wt or mouse adapted A/Aichi/2/1968 (H3N2) at 12 hpi with 36 or 22 dysreguated genes respectively [73]. This pattern of host gene regulation was host dependent and was reversed for the F103L and M106I single mutations in the human cells but again the low effect, in this case of the F103L mutation, was dominant in the F103L + M106I combination. The numbers of dysregulated genes due to HK-wt infection were comparable to that published for seasonal and pandemic H1N1 strains at 8 hpi in A549 cells (with 44 and 49 differentially regulated genes respectively) [74] or for HPAI H5N1 or H5N1 *cold adapted* vaccine (24 and 128 genes respectively) at 8 hpi [75]. The reduced effect on host gene expression by the HK-wt and F103L mutant was consistent with the maintenance of CPSF binding for these viruses in human cells and is supported by decreased host gene expression due to NS1 protein expression in human cell culture [76]. So in both human and mouse cells the double mutant was associated with a low effect on host gene regulation and was independent of CPSF binding in mouse cells but correlated with CPSF binding in human cells, suggesting that this pair of mutations may have modulating effects on host gene dysregulation in alternative hosts. Although we have not extended our analysis to the characterization of individual genes, only 3 genes were highly up regulated in both hosts for HK-wt and mutants that included IFN induced genes with tetratricopeptide repeats IFIT1 gi: 3434, IFIT1B gi: 439996, and IFIT2 gi: 3433 in addition to the ubiquitin peptidase USP41 gi: 373856. Both hosts also shared 2 genes that were down regulated to the greatest extent among HK-wt and all mutants; Mphosph6, M phase phosphoprotein 6 gi: 68533 involved in ribosomal RNA processing and Cdkn2aip CDKN2A interacting protein gi: 70925 involved in cell cycle regulation.

### Enhanced IFN antagonism and disease

In addition to human cells, each mutation also increased IFN antagonism *in vitro* in mouse cells to decrease IFN-β induction; 103L was more effective than 106I in this regard, reducing IFN to undetectable levels when present alone or in combination (with 106I) suggesting that the 103L mutation may provide a dominant ability to antagonize the IFN response (that supplements the incomplete ability of 106I to antagonize IFN induction) (Figure 3). Although the double 103L + 106I mutant did not induce IFN in mouse cells, it induced the same amount of IFN-β in mouse lungs as the consensus H5N1-NS1 103F+106M mutant that was attenuated due to the restriction of infection to bronchiolar tissues (Figure 2). The data is consistent with a model where H5N1-NS1 103F+106M virus infects the bronchiolar epithelium in the lung but induced inhibitory level of IFN that prevented alveolar infection and limited peak titers to 10^5^ pfu/g in contrast to the 103L+106I variant that produced 10^8^ pfu/g at 3 dpi in the lung due to the spread of infection to the alveoli in the presence of the same levels of IFN-β at 1 and 3 dpi (Figure 2). However the NS1 consensus sequence mutant, 103F+106M, was inhibited from further replication by the induced IFN-β and was cleared by day 7 in contrast to 103L+106I that continued to replicate, due to increased NS1 cytoplasmic abundance and/or activity, but that continues to induce IFN-β with an average yield of 1054 pg/g at 5 dpi (Figure 2). Thus lethal pathology at these doses may have been a function of extensive tissue infection (Figure 2 and 3) and high cytokine induction to induce hypercytokinemia of IFN-β that approximated the mouse LD_50_ intramuscular dose for type I IFN of 1,300 pg/g [77] indicating that these mice possessed toxic levels of IFN-β, that may contribute to the lethality associated with the 103L+106I induced pneumonia (Figures 1, 2 and 3). Although the *in vivo* data of enhanced IFN-β induction in the mouse lung (Figure 3b) appears to contradict the reduced IFN-β induction seen in mouse cells seen in Figure 3b, these findings are however consistent with the low level of human IFN-β induced by A/HK/156/1997 (H5N1) virus on infection of human A549 epithelium [78] versus the high IFN-β seen in humans infected with H5N1 viruses [79]. This may be due to the fact that other cell types are infected in the lung that induce high amount of IFN, such as shown for several H5N1 strains or their NS1 genes expressed in A/WSN/1933 (H1N1) reassortants that induced higher levels of cytokines including IFN-β [80]. Thus the high IFN-β levels produced in mouse lungs infected with the H5N1 NS1-wt virus may be due to the infection of other cell types in addition to epithelium in mouse lung such as immune cells including macrophages (Figure 3c). Thus IFN antagonism *in vitro* and *in vivo* may differ due to the extent of infection and the types of cells that are infected. However, further analysis of the roles of individual cell types in the infected mouse lung are required to explain the differences in IFN responses and pathology.

### Epistasis in NS1

We have previously shown that each of the F103L and M106I NS1 mutations in the HK virus increase virulence in the mouse without increasing IFN induction [15]. Further analysis of these mutations in the same HK1 NS1 gene but on the PR8 backbone showed increased virulence for each of the F103L and M106I mutations however virulence was not further increased in combination due to increased IFN-β production [13]. However we have shown here (Figure 3) and previously [13,15] that the IFN antagonism phenotypes of the 103L and 106I mutations are dependent on each other as well as the NS1 gene and virus backbones indicating that these mutations are subject to epistasis. However the F103L and M106I mutations in the H5N1 NS1 gene on the PR8 backbone resulted in gain-of-function seen as increased replication, virulence and IFN antagonism ([13], Figures 1, 2 and 3) indicating that the H5N1 NS1 gene possessed mutations that control the negative epistatic effects of this pair of mutations on the PR8 backbone [13]. This negatives epistasis may explain the previous observation of the A/HK/483/1997 (H5N1) virus that possesses the 103L+106I mutations and has extreme virulence for mice with of LD_50_ = 10 pfu whereas the L103F+I106M mutant had increased in virulence to result in LD_50_ = 0.032 pfu (the effect of each of the L103F or I106M mutations in isolation was not assessed), however the lethality of infectious dosages that are less than 1 suggests that the infectivity assays underestimated true mouse infectivity, thus complicating interpretation of these findings [16]. Epistasis may similarly explain the lack of effect of the 103S + 106I mutations on virulence when mutated back to consensus (S103F + I106M) in the hypervirulent variant of PR8 (hvPR8) virus in BALB/c mice (but with a 6 fold increase of virulence seen in MX1 mice) however the effect of each mutation in isolation was not assessed [81]. This is in contrast to the gain-of-function that the103S and 106I mutations provided in the standard PR8 strain (Figure 1e and f). The F103L + M106I mutations had originally been associated with increased IFN-β induction that correlated with a loss of CPSF binding by the H5N1 NS1 [67] however this phenotype is not seen for the 103S and 106I mutations of hvPR8 that similarly abrogates CPSF binding but without affecting IFN-β induction [81] indicating that the genetic context of these mutations is critical for their IFN antagonism phenotypes. Our observed IFN independent loss of function due to the 103L and 103I mutations in Vero cells was consistent with the aforementioned studies; however these mutations functioned to enhance IFN antagonism in IFN competent systems (Figures 3, 4 and 6) as well as [13,15].

### Human RIG-I binding

We also observed that RIG-I domain binding of the avian-type H5N1 NS1 protein possessing the consensus 103F+106M residues was defective and did not bind any domains, but the single 106I mutation restored binding to all 3 domains (CARD, helicase and RD) and 103L restored binding to 2 domains (CARD and helicase) and the combination bound 2 different domains (CARD and RD) (Figure 6). Thus the restoration of human RIG-I binding by the 103L and 106I mutations may contribute to the ability of the 1997 H5N1 viruses to infect humans [79,82]. We have not yet assessed the effect of these mutations on mouse RIG-I binding and further studies are needed to assess the role of RIG-I binding in modulating IFN antagonism in human and mouse cells in addition to the roles for TRIM25 and RIPLET binding in mouse adaptation [34]. In addition to the demonstration that F103L and M106I mutations are under positive selection for human IAV H3N2 in the mouse, they were also independently selected in avian H9N2 and H6N1 lineages in Asia. Significantly these mutant NS1 genes can transfer to additional subtypes by reassortment, including H5N1, H6N1 and H7N9 viruses, which can infect multiple avian species and transmit to humans illustrating the potential for these mutations to promote host switching among multiple species (Figure 9). We demonstrate that both of these mutations provide gain-of-function to increased cytoplasmic expression and possibly altered host factor function to enhance interferon antagonism. These mutations enhance virulence in the mouse model of interstitial pneumonia by mediating the productive infection of alveolar tissue which provides a model for examining enhanced infection and disease of avian 1997 H5N1 and H7N9 viruses in humans that are likewise associated with severe interstitial pneumonia with associated cytokinemia [5,6] (as also seen in the mouse model (Figures 1, 2 and 3)). The first human H6N1 influenza infection possessed the NS1 F103L mutation further linking this mutation with host switching.

## Conclusion

We hypothesize that 103L and 106I increased the IFN antagonism properties of H5N1 NS1 in mice by increasing the expression and trafficking of NS1 into the cytoplasm as well as increased host factor binding such as RIG-I to mediate enhanced functional interactions with the IFN sensing and IFN effecter functions of the host.

## Abbreviations

CPSF30: *Cleavage and polyadenylation specificity factor 30*; F2F3: *F2 and F3 zinc finger domain*; RIG-I: *Retinoic acid*-*inducible* gene 1; RD: *Regulatory domain*; RTHS: Bacterial reverse 2-hybid system; pfu: *Plaque forming units*; LD50: *Median lethal dose*; LPAI: *Low pathogenic avian influenza*; HPAI: *High pathogenic avian influenza.*

## Competing interests

All of the authors declare that they have no competing interests with respect to the publication of this manuscript.

## Author contributions

Conceived and planned experiments: EGB, SKD, EM, AT. Performed experiments: SKD, EM, NEF, MS, JP, JJ. Analyzed the data: SKD, EM, NEF, MS, JP, MP, EGB, AT. Supplied reagents: EGB, AT, MP. Wrote the paper: SKD, EGB, AT, MS. Edited the paper: SKD, EM, NEF, MP, AT, MS, JP, JJ, EGB. All authors read and approved the final manuscript.

## Supplementary Material

Additional file 1: Table S1 List of primers used for mutagenesis of NS1 genes. **Table S2.** List of NS1 gene accession numbers for phylogenic tree shown in Figure 9. **Figure S1.** Bacterial Reverse two-hybrid Assessing the interaction of NS1 with the CARD, helicase and RD domains of Rig-I. **Figure S2.** Lack of nonspecific binding of recombinant H5N1 NS1 protein variants to the antibody bead matrix in pull-down assays. **Figure S3.** Plots of host gene expression in mouse cells relative to mock infected cells for rHK-NS1-wt, -103L, -106V, 106I, and -103L + 106I mutants. **Figure S4.** Full phylogenic tree of the NS1 amino acid sequences of the viruses used to make Figure 9. Click here for file
