# Supplementary material for: Influenza A/Hong Kong/156/1997(H5N1) virus NS1 gene mutations F103L and M106I both increase IFN antagonism, virulence and cytoplasmic localization but differ in binding to RIG-I and CPSF30
Source: Virol J. 2013 Jul 25;10:243. doi: 10.1186/1743-422X-10-243 (PMC3733596; doi:10.1186/1743-422X-10-243)

Supplementary Tables S1-S2 and Figures S1-S4 for

Influenza A virus A/Hong Kong/156/1997(H5N1) NS1 gene mutations F103L and M106I both function to increase virulence and cytoplasmic localization but differ in binding to RIG-I and CPSF30

Samar K. Dankar[1,2¶], Elena Miranda[3¶], Nicole E. Forbes[1,2], Martin Pelchat[1], Ali Tavassoli[3,4], Mohammed Selman[1,2], Jihui Ping[1,2], Jianjun Jia[1], and Earl G. Brown[1,2,*]

Supplementary Tables:

Table S1. List of primers used for mutagenesis of NS1 genes. The name for each primer indicates the NS1 gene source as well as the mutation and sense of each primer.

Table S2. List of NS1 gene accession numbers for phylogenic tree shown in Fig. 9.

Table S1 List of Primers for mutagenesis of NS1 genes. The name for each primer indicates the NS1 gene source as well as the mutation and sense of each primer.

1. **H5N1-L103F-forward**: GAC TGG TTC ATG CTC ATT CCC AAG,
2. **H5N1-L103F-reverse**: CTT GGG AAT GAG CAT GAA CCA GTC
3. **H5N1-I106M-forward**: CTG GTT AAT GCT CAT GCC CAA GCA G
4. **H5N1-I106M-reverse**: CTG CTT GGG CAT GAG CAT TAA CCA G
5. **H5N1-L103F+I106M forward**: TCA AGG GAC TGG TTC ATG CTC ATG CCC AAG CAG
6. **H5N1-L103F+I106M-reverse**: CTG CTT GGG CAT GAG CAT GAA CCA GTC CCT TGA
7. **PR8-S103F forward**: GGA CTG GTT CAT GCT CAT ACC CAA G
8. **PR8-S103F-reverse**: CTT GGG TAT GAG CAT GAA CCA GTC C,
9. **PR8-I106M-forward**: GTC CAT GCT CAT GCC CAA GCA GAA AG
10. **PR8-I106M-reverse**: CTT TCT GCT TGG GCA TGA GCA TGG AC
11. **PR8-S103F+I106M-forward**: AGG GAC TGG TTC ATG CTC ATG CCC AAG CAG A
12. **PR8-S103F+I106M-reverse**: TCT GCT TGG GCA TGA GCA TGA ACC AGT CCC T
13. **HK-F103L-forward:** GGA CTG GTT CAT GCT AAT GCC CAA G
14. **HK-F103L-reverse:** CTT GGG CAT TAG CAT GAA CCA GTC C
15. **HK-M106I-forward:** ACT GGT TCA TGC TAA TTC CCA AGC AGA AAG TG
16. **HK-M106I-reverse:** CAC TTT CTG CTT GGG AAT TAG CAT GAA CCA GT
17. **HK-F103L+M106I-forward**: GAC TGG CTC ATG CTA ATT CCC AAG
18. **HK-F103L+M106I-reverse**: CTT GGG AAT TAG CAT GAG CCA GTC

Table S2 List of accession number of NS1 sequences used in Fig S4 and Fig 9.

Viruses	Accession Number
A/chicken/Korea/MS96-CE6/1996	ACZ47499
A/pheasant/Hong Kong/SSP44/2002	BAJ18081
A/chicken/Guangxi/KMI/99	AAX76911
A/Hong Kong/483/1997	ACZ47223
A/Chicken/Beijing/1/94	AF156480_2
A/duck/Hong Kong/702/1979	ACD12177
A/chukkar/Shantou/3809/2003	ABU97331
A/Thailand/271/2005	ABK57102
A/quail/Shantou/149/2003	ABM46367
A/turkey/Ohio/313053/2004	ACD88664
A/mallard/New York/6874/1978	AAA43545
A/swine/Iowa/A01203943/2012	AGC96258
A/avian/Saudi Arabia/910135/2006	ACY80171
A/chicken/Hong Kong/915/97	AF098573_1
A/duck/Shantou/5753/2001	ABV47624
A/chicken/Middle East/ED-1/1999	GU053205
A/mallard/California/2531P/2011	AGE08728
A/chicken/HongKong/BD90/03	AAU11227
A/chukkar/Shantou/1530/2005	ABU97433
A/swine/Iowa/A01202529/2011	AFN66921
A/baikal teal/Xianghai/421/2011	AFZ15771
A/swine/Korea/CAS05/2004	ACE78049
A/quail/Shantou/22124/2005	ABU97451
A/chicken/Tunisia/12/2010	ADX99482
A/chicken/Korea/121/2007	AEO27729
A/chicken/Osaka/aq58/2001	BAF46463
A/goose/MN/5733-1/1980	ABB88395
A/avian/Saudi Arabia/910134/2006	ACY80169
A/Hong Kong/486/97	AF115289_1
A/duck/Hong Kong/7/1975	ABB88261
A/swine/Iowa/2/1987	ABX58651
A/quail/Shantou/2061/2000	ABM46311
A/Shanghai/1/2013	EPI_ISL_138737 (Nucleotide)
A/chicken/Iran/N101/2011	AGC96430
A/mallard/Minnesota/186/1999	DQ021595
A/chicken/Korea/97/2007	AEO27727
A/chicken/Korea/01310/2001	AFM47145
A/Shandong/1/2009	AEO89130
A/quail/yunnan/092/2002	ABD23025
A/Duck/Shantou/1043/00	AAP49170.1
A/mallard/New Zealand/275-15/2005	ADR65384
A/chicken/India/100071/2008	AFK10228

A/chicken/Korea/S1/2003	AAV65821
A/chicken/Dubai/463/2003	ABM21923
A/chicken/Heilongjiang/u/1998	AEB71197
A/turkey/Israel/1209/2003	ABG76045
A/Chicken/Guangdong/11/97	AF508716_1
A/swine/Korea/S452/2004	AAV68026
A/Chicken/Hong Kong/y388/97	AF098570_1
A/quail/Dubai/301/2000	ABM21911
A/chicken/Banten/Pdgl-Kas/2004	ADB08029
A/chicken/Dubai/338/2001	ABM21917
A/muscovy duck/Vietnam/LBM227/2012	BAM84139
A/swine/Tennessee/2/1978	ABW71508
A/chicken/Shandong/6/96	AAY52676
A/chicken/Iran/68/2006	ADO39891
A/swine/Thailand/CU-PS73/2010	AEB80454
A/chicken/Guangxi/6/2000	ABV31863
A/chicken/Emirates/R66/2002	ADP69278

Supplementary Figures:

Figure S1 Mechanism of the Bacterial Reverse Two-hybrid Assay (RTHS) for the interaction of NS1 with the CARD, helicase and RD domains of human RIG-I. (a) Interaction of NS1 with X (CARD, helicase or RD) results in the formation of a 434/P22 functional repressor that prevents expression of the downstream reporter genes, inhibiting survival of the host strain on selective media. (b) If the two target proteins do not interact, the 434/P22 repressor is not formed, and so the host strain survives on selective media. Figure modified from Miranda et al (2011).

Figure S2 Lack of nonspecific binding of recombinant H5N1 NS1 protein variants to the antibody bead matrix in pull-down assays. The input NS1 and CPSF30 F2F3 proteins are shown and labelled in "input" relative to the pull down proteins in the absence of CPSF30 F2F3 labelled as "no CPSF30 F2F3 FLAG". To test nonspecific binding of NS1 proteins to the antibody bead matrix, S^{35} labelled NS1 protein mixed with protein G beads and FLAG antibody in the absence of the CPSF30 F2F3 fragment showing a lack of binding to the pull-down matrix in the absence of CPSF30. Pull down of H5N1-NS1-wt-L103F +I106Mby CPSF30 F2F3 was included as a positive control. NS1 proteins and CPSF F2F3 are indicated on each autoradiogram.

Figure S3. Plots of host gene expression in mouse cells relative to mock infected cells for rHK-NS1-wt, -103L, -106V, 106I, and -103L + 106I mutants.
a. A box plot of dysregulated genes from Fig 7a shown relative to the gene expression of HK-wt relative to mock infected M1 cells for the indicated mutants. b. Linear plot of the relative gene expression relative to HK-wt was plotted with the line of best fit for dysregulated genes in mouse M1 cells shown in figure 7a. The slope of each line is indicated relative to the HK-wt line with slope = 1 against itself.

Figure S4. Full phylogenic tree of the NS1 amino acid sequence of the viruses used to make Figure 9.

Figure S1. Mechanism of the Bacterial Reverse Two-hybrid Assay (RTHS) for the interaction of NS1 with the CARD, helicase and RD domains of human RIG-I.

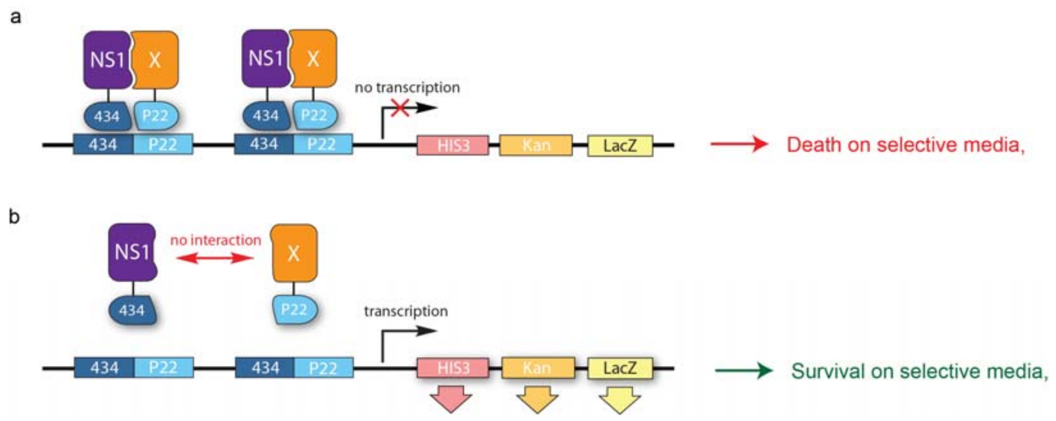

Figure S2. Lack of nonspecific binding of recombinant H5N1 NS1 proteins to antibody bead matrix in pull-down assays in the absence of CPSF30 F2F3-FLAG.

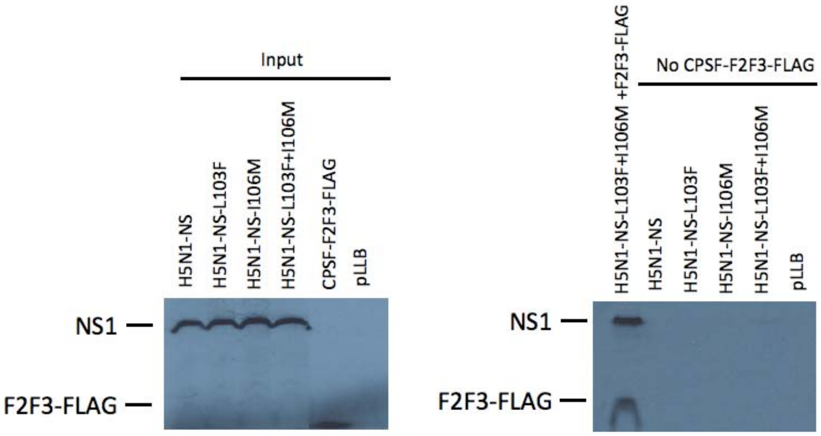

Fig S3. Plots of host gene expression in mouse cells relative to mock infected cells for rHK-NS1-wt, -103L, -106V, 106I, and -103L + 106I mutants.

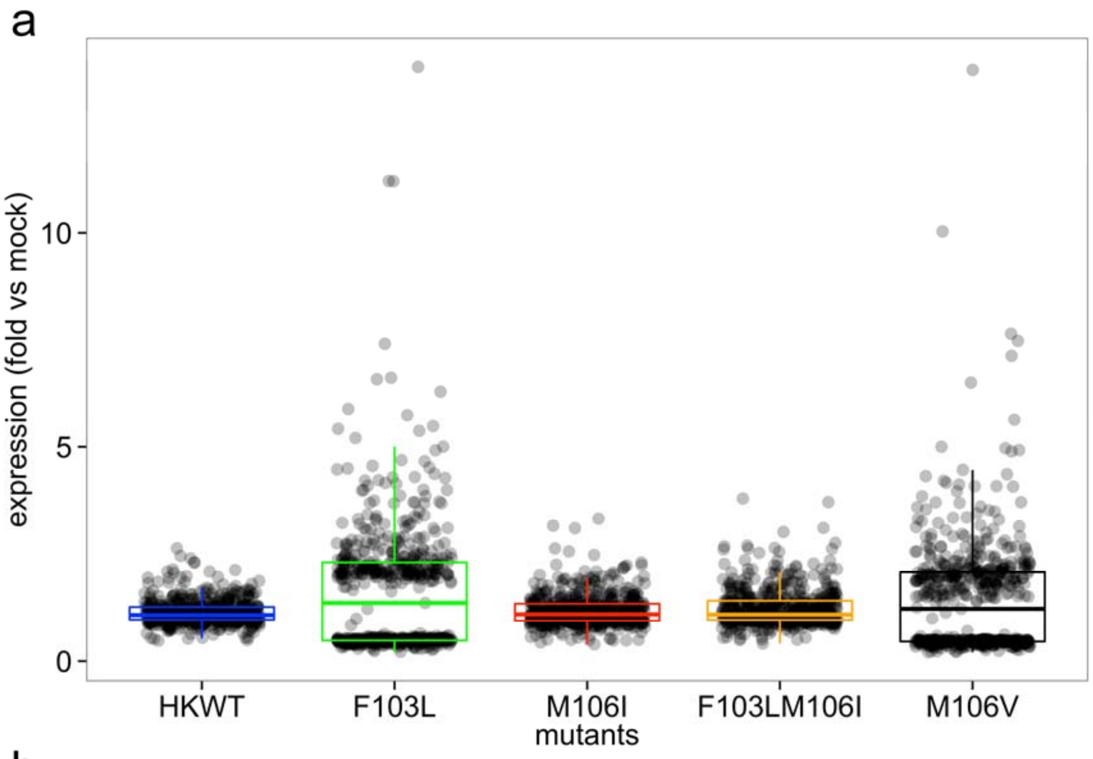

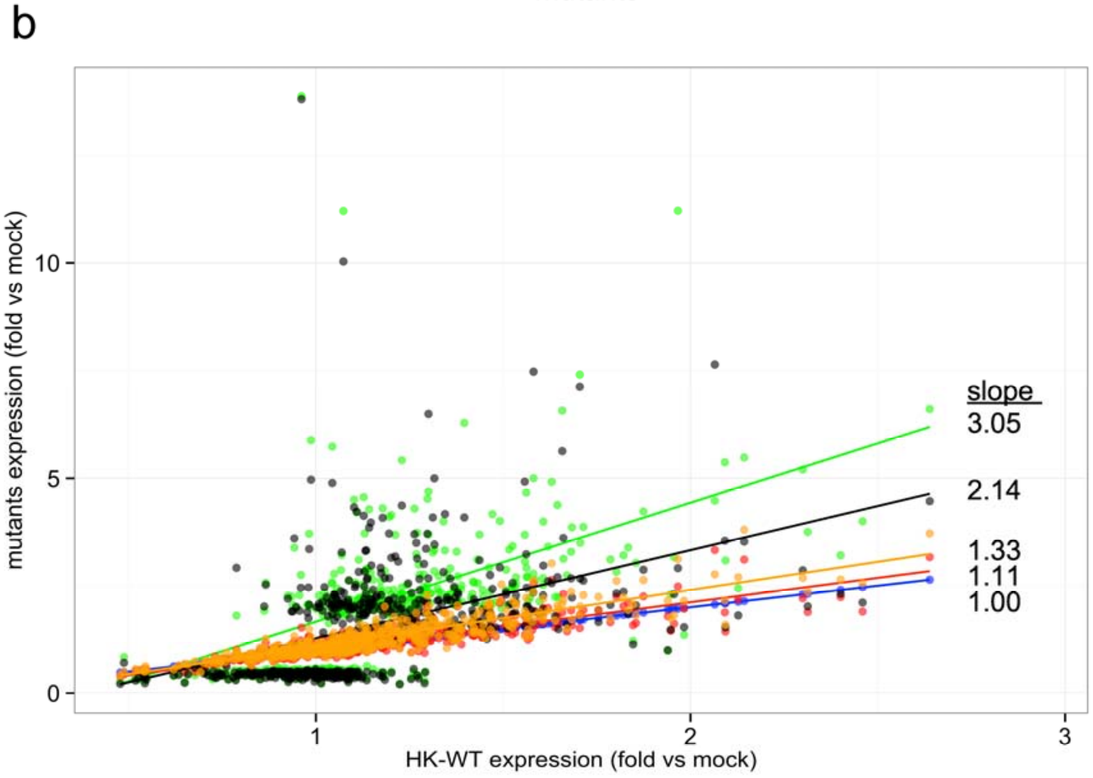

Figure S4. Full phylogenic tree of the NS1 amino acid sequences of the viruses used to make Figure 9.

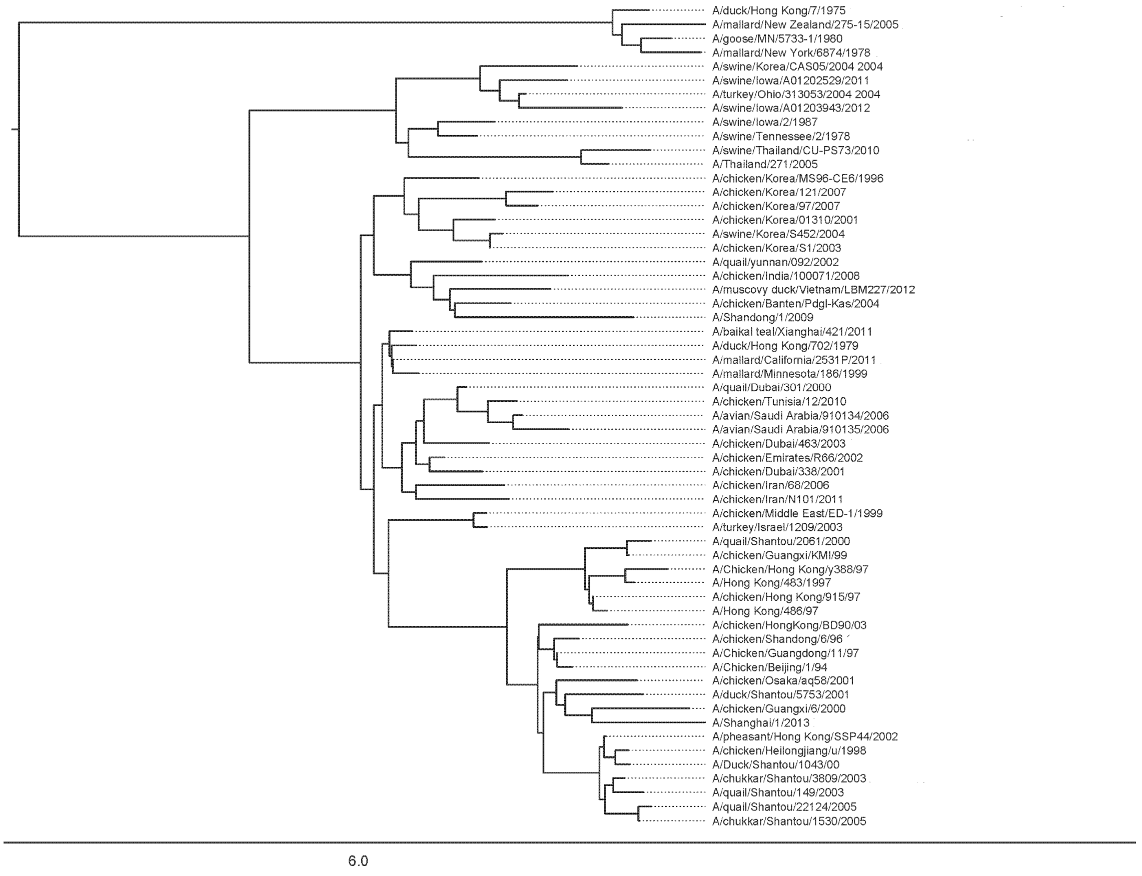

Supplement: Additional file 1: Table S1 — List of primers used for mutagenesis of NS1 genes. Table S2. List of NS1 gene accession numbers for phylogenic tree shown in Figure 9. Figure S1. Bacterial Reverse two-hybrid Assessing the interaction of NS1 with the CARD, helicase and RD domains of Rig-I. Figure S2. Lack of nonspecific binding of recombinant H5N1 NS1 protein variants to the antibody bead matrix in pull-down assays. Figure S3. Plots of host gene expression in mouse cells relative to mock infected cells for rHK-NS1-wt, -103L, -106V, 106I, and -103L + 106I mutants. Figure S4. Full phylogenic tree of the NS1 amino acid sequences of the viruses used to make Figure 9. [file 1743-422X-10-243-S1.pdf]
